# Opening a window to skin biomarkers for diabetes stage with optoacoustic mesoscopy

**DOI:** 10.1038/s41377-023-01275-3

**Published:** 2023-09-18

**Authors:** Hailong He, Nikolina-Alexia Fasoula, Angelos Karlas, Murad Omar, Juan Aguirre, Jessica Lutz, Michael Kallmayer, Martin Füchtenbusch, Hans-Henning Eckstein, Annette Ziegler, Vasilis Ntziachristos

**Affiliations:** 1 Institute of Biological and Medical Imaging, Helmholtz Zentrum München, Neuherberg, Germany; 2 Chair of Biological Imaging at the Central Institute for Translational Cancer Research (TranslaTUM), School of Medicine, Technical University of Munich, Munich, Germany; 3 Department for Vascular and Endovascular Surgery, Klinikum rechts der Isar, Technical University of Munich (TUM), Munich, Germany; 4 DZHK (German Centre for Cardiovascular Research), partner site Munich Heart Alliance, Munich, Germany; 5 Diabetes Center at Marienplatz, Munich, Germany; 6 Forschergruppe Diabetes e.V., Helmholtz Zentrum München, Neuherberg, Germany; 7 Institute of Diabetes Research, Helmholtz Zentrum München, Neuherberg, Germany

**Keywords:** Imaging and sensing, Photoacoustics

## Abstract

Being the largest and most accessible organ of the human body, the skin could offer a window to diabetes-related complications on the microvasculature. However, skin microvasculature is typically assessed by histological analysis, which is not suited for applications to large populations or longitudinal studies. We introduce ultra-wideband raster-scan optoacoustic mesoscopy (RSOM) for precise, non-invasive assessment of diabetes-related changes in the dermal microvasculature and skin micro-anatomy, resolved with unprecedented sensitivity and detail without the need for contrast agents. Providing unique imaging contrast, we explored a possible role for RSOM as an investigational tool in diabetes healthcare and offer the first comprehensive study investigating the relationship between different diabetes complications and microvascular features in vivo. We applied RSOM to scan the pretibial area of 95 participants with diabetes mellitus and 48 age-matched volunteers without diabetes, grouped according to disease complications, and extracted six label-free optoacoustic biomarkers of human skin, including dermal microvasculature density and epidermal parameters, based on a novel image-processing pipeline. We then correlated these biomarkers to disease severity and found statistically significant effects on microvasculature parameters as a function of diabetes complications. We discuss how label-free RSOM biomarkers can lead to a quantitative assessment of the systemic effects of diabetes and its complications, complementing the qualitative assessment allowed by current clinical metrics, possibly leading to a precise scoring system that captures the gradual evolution of the disease.

## Introduction

Diabetes mellitus is a complex metabolic disease with increasing worldwide prevalence, leading to several health complications and aggravating healthcare costs^1,2^. The disease affects the macro- and the microvasculature of several organs, including the heart, brain, lower limbs, retinas, peripheral nerves, kidneys, and skin^1–4^. In the skin, diabetes-induced microvasculature alterations indicate an adverse disease prognosis, as they compromise tissue perfusion and oxygenation, as well as skin integrity, which can lead to cutaneous infections^3,5–8^, neuropathy with loss of sensation, ulcerations, and other comorbidities^3,5–7^. These microvascular changes may also indicate cardiovascular complications such as coronary artery disease (CAD), carotid artery disease, and peripheral arterial disease (PAD)^9–11^ and occur early in the development of diabetes^3,4,12^. Therefore, assessment of skin microvasculature could lead to a novel means of monitoring diabetes onset and the progression of associated vascular complications, allowing quantification of the true burden of the disease on the vascular system rather than disease course predictions offered by risk factors.

Currently, the characterization of diabetes stage and its complications in patients relies on the assessment of clinical symptoms and signs. In many instances, questionnaires and scoring systems are employed to assess the presence and quality of peripheral neuropathy symptoms and signs such as neuropathic pain, decreased perception of light touches or muscle weakness, and can be combined with clinical tests assessing pathophysiological parameters^5,6^. Such assessments may offer subjective readings, are time-consuming, and generally evaluate the progression of the disease and its complications at infrequent intervals, during which diabetes has advanced significantly enough to yield large pathophysiological changes that present as clinical symptoms relating to loss of function of different organs and/or pain.

Disease manifestations in the skin microvasculature could serve as a means to observe the multi-systemic effects of diabetes and its complications in a quantitative fashion and possibly lead to finer and more detailed information in the course of the disease based on gradual changes that are not perceivable as clinical symptoms. Skin as the largest and most easily accessible organ could serve as a window for diabetes microangiopathy and staging of the disease. However, routine assessment of epidermal features and dermal microvasculature requires a method appropriate for safe, longitudinal, direct, and non-invasive measurements. Located under the highly-scattering epidermis, dermal vasculature is not generally accessible to optical microscopy methods, such as confocal or two-photon microscopy^13,14^. Other methods, such as high-frequency ultrasound^15–18^, hyperspectral imaging^19,20^, nailfold capillaroscopy^21,22^, and optical coherence tomography (OCT)^23,24^, have various advantages. However, in general, these technologies do not provide sufficient resolution, contrast, and/or penetration depth to visualize skin microvasculature and hence application has mostly been restricted to differentiating patients with diabetes from healthy subjects^23,24^. Although certain methods have demonstrated microvascular variations between individuals with and without diabetes, to the best of our knowledge, none of these methods have been utilized to classify disease progression or its complications or employed to examine the correlation between microvascular imaging biomarkers and diabetic complications. Such information is an important goal for an imaging method, as it would address a current gap in diabetes research associated with disease staging. Currently, only crude, and infrequent assessments of disease complications known to affect the quality of delivered healthcare are done^25,26^, as elaborated in the discussion section of this paper.

It has been demonstrated that OCT angiography (OCTA) and ultra-wideband raster scan optoacoustic mesoscopy (UWB-RSOM) both have the potential to indirectly detect vasculature by capturing small signal variations resulting from micro-flows, thereby presenting non-invasive approaches for evaluating cutaneous changes in human skin microvasculature^23,27–33^. In this work, we have chosen to focus on UWB-RSOM due to certain key features. UWB-RSOM is a notably robust method for visualizing deep dermal microvasculature features (up to 1.5 mm deep) and this ability will enable our search to identify novel biomarkers of skin microangiopathy in diabetes. Furthermore, RSOM offers detection with an enhanced signal-to-noise ratio due to the generation of optoacoustic signals within blood vessels primarily. This phenomenon allows for optimal microvascular imaging of the skin, while retaining high contrast due to the relatively high absorption of hemoglobin at the wavelength of 532 nm^28,34–39^. Furthermore, it was important to obtain highly detailed cross-sectional images of human skin at high penetration depth because these images would offer valuable information for diagnosis, treatment monitoring, and translational research from understanding skin physiology and pathology and indeed UWB-RSOM can provide such images to a depth of ~1.5 mm.

We therefore employed UWB-RSOM to evaluate the effect of diabetes on skin, offering the first in vivo insights on the relation of dermal and epidermal features and diabetes complications. To this end, we performed measurements on 143 subjects including healthy individuals without diabetes and participants with diabetes. The diabetic group comprised of participants with previously diagnosed diabetes and no other symptoms, participants with diabetes and peripheral neuropathy and participants with diabetes and macrovascular atherosclerotic complications. We were specifically interested in exploring how diabetes progressed, as evidenced in this study by the presence of different complications, effects on different dermal and microvascular components and whether it could be correlated to any of the calculated skin features. We postulated that we could employ image analysis techniques to detect and quantify RSOM skin features associated with the stage of diabetes mellitus, and this would be an improvement to the currently done characterization based on clinical symptoms and comorbidities.

## Results

### RSOM imaging and biomarker computation

**Fig. 1:**
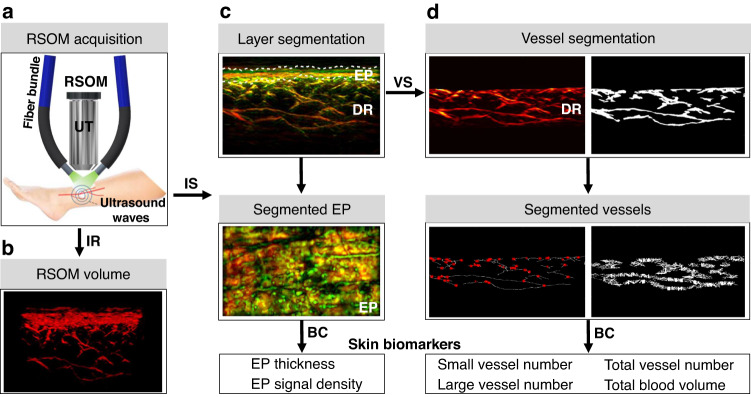
Computation pipeline of skin biomarkers from RSOM images. **a** Schematic of the RSOM system employed for skin measurements, comprising two fiber bundles for illumination and a high frequency ultrasound transducer (UT) that was raster scanned over the skin surface. RSOM signals are recorded on the pretibial area of the lower extremities of both healthy volunteers and participants with diabetes, after which volumetric image reconstruction (IR) is performed. **b** A reconstructed RSOM volume image. The volumetric RSOM image is segmented (IS) to identify the epidermis layer and dermal vasculature, which are used to subsequently compute biomarkers. **c** Segmentation of the cross-sectional RSOM image into the epidermis (EP) and dermis (DR) layers of the skin (white dashed lines). The EP thickness and EP signal density biomarkers were computed from the segmented EP layers in the RSOM images. **d** Vessel segmentation of the segmented DR layer of the skin. The numbers of vessel branches and vessel diameters were automatically calculated; the red dots indicate positions of vessel branches. The segmented vessels in the DR were used to calculate the vessel numbers and total blood volume biomarkers. IR image reconstruction, IS image segmentation, VS vessel segmentation, BC biomarker computation, scale bar = 500 µm

To enable quantitative analysis of diabetic skin microvascular features, we collected RSOM measurements from 95 participants with diabetes and 48 volunteers without diabetes and developed an imaging analysis pipeline to compute skin biomarkers (Fig. 1). RSOM illuminated the surface of the skin over the pretibial area at 532 nm and scanned with an ultrasound transducer with bandwidth from 10 MHz to 120 MHz and central frequency of 50 MHz over a 4 × 2 mm^2^ field of view (FOV, Fig. 1a, see RSOM imaging system in “Methods” section). Three-dimensional RSOM images (Fig. 1b, see image reconstruction in “Methods” section) were reconstructed over two frequency bands within the 120 MHz bandwidth employed. Band-selected reconstructions implicitly segmented vessels of different sizes; larger vessels (40–150 µm) are seen in the 10–40 MHz band, whereby smaller vessels (<10–40 µm) are seen in the 40–120 MHz band. Vessels seen in the two different bands are color-coded in the rendered images (red: larger vessels; green: smaller vessels) so that finer vasculature is highlighted in the presence of larger vessels (Fig. 1c). To quantify the differences observed by visual inspection of the RSOM images, as well as to extract relevant label-free RSOM biomarkers, we developed and validated a RSOM image analysis pipeline including two segmentation methods (see layer and vasculature segmentation section in “Methods” section). Briefly, a layer segmentation algorithm based on graph theory and dynamic programming^40^ identified and separated the epidermis and dermis, as visually marked on the images (Fig. 1c, white dashed lines; see layer segmentation section in “Methods” section and Fig. S2). The second method employed a vessel segmentation algorithm^41^ to identify and quantify vascular structures in the dermis layer (Fig. 1d; see vessel segmentation section in “Methods” section and Fig. S3). Quantification included the computation of the vessel number and the diameter of the different vessels identified. Validation of the segmentation approach was performed by comparing the RSOM computed biomarkers of mice skin with histological analysis (see validation section in “Methods” section and Figs. S2 and S4).

Based on this RSOM image analysis pipeline, we computed and differentially analyzed six RSOM image features (see biomarker computation section in “Methods” section): (1) the total number of small vessels (with diameters <40 µm; 40–120 MHz band) in the dermal layer; (2) the total number of large vessels (with diameters >40 µm; 10–40 MHz band) in the dermal layer; (3) the total vessel number in the dermal layer; (4) the total blood volume in the dermal layer, computed as ratio of the total number of volume elements (voxels) occupied by the segmented vessels over the total number of voxels in the image; (5) the epidermal thickness, and (6) the epidermal signal density. The selection of small vs. large vessels based on the 40 µm cut-off value was explained (see “Methods” section) and a more detailed analysis based on a finer vessel classification is shown in Fig. S5.

### Skin microvasculature differences between healthy volunteers and participants with diabetes

**Fig. 2:**
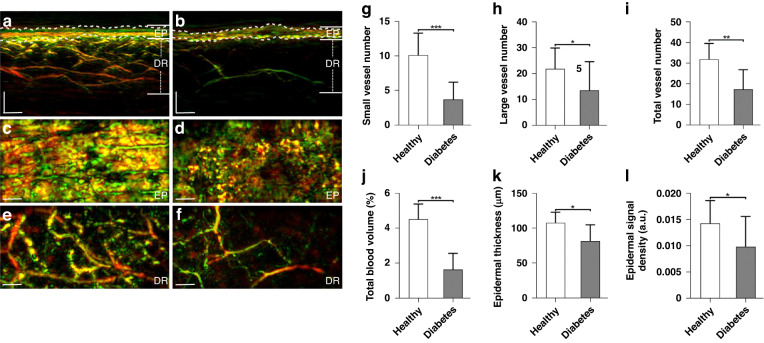
Skin imaging of the lower extremities (distal pretibial region) of healthy volunteers and participants with diabetes using clinical RSOM. **a** Sectional RSOM image of healthy skin. **b** Sectional RSOM image of skin from a participant with diabetes but without neuropathy. MIP images of the EP and DR layers in the coronal views corresponding to (**a**, **b**) are displayed in (**c**, **d**) and (**e**, **f**), respectively. **g**–**I** Comparisons of computed biomarkers between healthy volunteers and participants with diabetes. **g** Total number of small vessels (with diameter ≤40 µm) in DR layer. **h** Total number of large vessels (with diameter >40 µm) in DR layer. **i** Total numbers of vessels in DR layer. **j** Total blood volume of the DR vasculature. **k** Average thicknesses of the EP layers. **l** Signal densities of the EP layers. The healthy volunteer (control) group had a population of 48, while the group of participants with diabetes had a population of 95. *P < 0.05, **P < 0.01, and ***P < 0.001, respectively. Scale bar = 500 µm. EP epidermis, DR dermis

We first analyzed RSOM skin images of the lower extremities (distal pretibial area) of 95 participants with diabetes and 48 volunteers without diabetes, where the characteristics of study participants are listed in Table 1 (see data grouping section in “Methods” section). Inspection of 2-band RSOM images of the skin (Fig. 2) visually exemplifies differences between a healthy volunteer and a participant with diabetes mellitus. Figure 2a depicts an image from a 36-year-old female volunteer without diabetes, while Fig. 2b shows the corresponding image from a 42-year-old male participant with diabetes. The images are rendered as maximum intensity projections (MIP) of the entire volume scanned and depict the epidermal (EP) and dermal (DR) layers, reaching a depth of ~1.5 mm. The typical RSOM appearance of healthy skin shows a dense signal from the epidermis layer (coronal view, Fig. 2c, d) and a vascular network in the dermal layer (coronal view, Fig. 2e) that comprises several blood vessels of various diameters. Conversely, the dermal vessel density (coronal view, Fig. 2f) is far lower in the participant with diabetes mellitus compared to the healthy volunteer, a finding that is confirmed by three-dimensional skin visualizations (see Suppl. Movie. 1 and 2). Due to the loss of fine dermal vasculature, the diabetic skin exhibits a characteristic high-contrast boundary between the epidermal and dermal layers that is not present in the healthy skin.

**Table 1:** Characteristics of study participants

	Participants with diabetes mellitus (DM) (95)	Participants with DM and no complications (45)	Participants with DM, neuropathy and no ASCVD (25)	Participants with DM, neuropathy and ASCVD (25)	Healthy volunteers (48)	*P*-value
Age (years)	68 ± 12	63 ± 19	70 ± 10	76 ± 7	64 ± 13	ns
Disease duration (years)	20 ± 16	12 ± 11	27 ± 17	23 ± 16	0	ns
Sex (male/female)	42/56	24/19	9/18	6/19	27/21	n/a
BMI (kg/m²)	27 ± 8	27 ± 6	30 ± 8	28 ± 4	26 ± 7	ns
Diabetes type (1/2)	21/74	7/36	11/14	3/22	n/a	n/a
NSS	4 ± 4	0	4 ± 4	7 ± 2	n/a	n/a
NDS	4 ± 4	0	4 ± 4	6 ± 3	n/a	n/a
HbA1c^a^ (%)	7.1 ± 1.1	7.1 ± 1.6	7.2 ± 0.7	6.9 ± 0.9	n/a	n/a

Data is presented as the mean ± SD unless stated otherwise. Participants with diabetes (*n* = 95) were further divided into three subgroups: Participants with DM and no complications [*n* = 45, participants with diabetes, but without neuropathy and without atherosclerotic cardiovascular disease (ASCVD)]; Participants with DM, neuropathy, and no ASCVD (*n* = 25, participants with diabetic neuropathy and without ASCVD); Participants with DM, neuropathy and ASCVD [*n* = 25, participants with diabetic neuropathy and ASCVD/PAD]

*DM* diabetes mellitus, *ASCVD* atherosclerotic cardiovascular disease, *PAD* peripheral arterial disease, *BMI* Body Mass Index, *NSS* Neuropathy Symptom Score, *NDS* Neuropathy Disability Score, *HbA1c* glycated hemoglobin, *ns* not statistically significant, *n/a* not applicable

^a^HbA1c values were not recorded for the 27 participants with diabetes and without complications

A next step was to examine the correlation of the six computed biomarkers (see Fig. 1c, d) to diabetes status (Fig. 2g–l). We found that the mean number of small vessels (Fig. 2g) was ~2.8 times less in participants with diabetes than in the volunteers without diabetes (3.45 ± 2.62 vessels versus 9.78 ± 3.41 vessels). A Mann–Whitney *U*-test showed statistically significant differences between the mean number of small vessels in healthy vs. diabetic subjects (*P* < 0.001). The mean number of large vessels (Fig. 2h) was about 1.5 times less in the diabetic compared to the healthy group (13.39 ± 11.30 vessels versus 20.34 ± 8.32 vessels, *P* < 0.05). This suggests that the systemic impacts of diabetes on vasculature are more prominent in small vessels than in larger vessels. These results were corroborated by analyzing the full band RSOM image (Fig. 2i), revealing the total vessel number in the volume examined. The total number of vessels was found to be 16.87 ± 9.30 for diabetic participants vs. 30.12 ± 9.87 for healthy volunteers (*P* < 0.01).

The total blood volume in the DR layer was also markedly different between the diabetic and healthy groups (Fig. 2j), with values of 1.58 ± 0.90% and 4.21 ± 1.10%, respectively. The Mann–Whitney *U*-test here also showed significant differences between the healthy and diabetic subjects (*P* < 0.001). Analysis of the EP layer also demonstrated statistically significant changes between the two groups. The mean values of epidermal thickness were 105.27 ± 17.04 µm for healthy volunteers and 81.03 ± 23.06 µm for participants with diabetes (Fig. 2k) with *P* < 0.05. Likewise, the signal density of the EP layer (Fig. 2l) in the reconstructed segmented volume, which contains contributions from melanin and capillaries, was markedly lower in the participants with diabetes than in the healthy volunteers (*P* < 0.05).

The relation between RSOM features and age (see Fig. S6), disease duration (see Fig. S7), body mass index (see Fig. S8) and HbA1c values (see Fig. S9) were investigated and shown in the supplementary results. We found that these parameters did not show obvious correlation with the RSOM biomarkers and did not significantly influence the outcome of our study. We have computed the Spearman correlation value between age/disease duration /HbA1c /BMI with the vascular biomarkers TBV (Total blood volume) and SVN (Small vessel number), which showed no significant correlation as presented in supplementary Table I. In addition, we also applied multivariate logistic regression analysis to compute the statistics of the two biomarkers TBV and SVN with adjustment of age/disease duration /Hb1Ac /BMI, which also revealed no significant association between these vascular biomarkers and characteristics of the participants (supplementary Table I). There were no significant differences in age, Hb1Ac and BMI values between the participant groups with type 1 or type 2 diabetes (supplementary Table V).

### Quantification of RSOM biomarkers in diabetic neuropathy

**Fig. 3:**
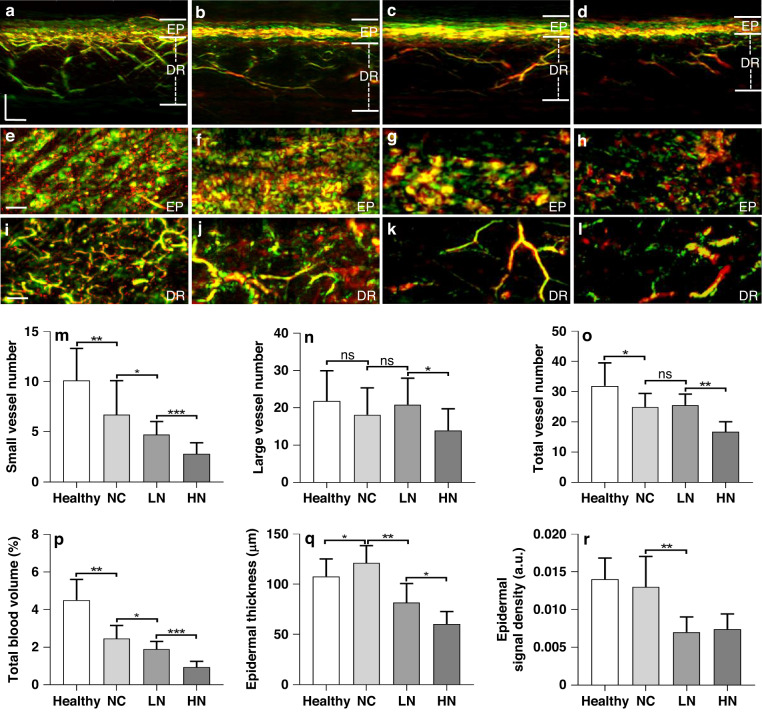
Quantification of RSOM features in participants with diabetic neuropathy. The diabetic participants were grouped as follows: NC (*n* = 45), participants and no complications; LN (*n* = 13), participants with diabetes and low score neuropathy (1 ≤ NDS ≤ 5 or 1 ≤ NSS ≤ 5); HN (*n* = 12), participants with diabetes and high score neuropathy (NDS > 5 or NSS > 5). **a** Sectional RSOM image of healthy skin. **b** Sectional RSOM image of skin from a participant with diabetes but without neuropathy. **c** Sectional RSOM image of skin from a participant with diabetes and low score neuropathy (NDS: 3, NSS: 3). **d** Sectional RSOM image of skin from a participant with diabetes and high score neuropathy (NDS: 9, NSS: 9). MIP images of the EP and DR layers in the coronal views corresponding to (**a**–**d**) are displayed in (**e**–**h**) and (**i**–**l**) respectively. **m**–**r** Comparisons amongst the four groups of participants with diabetes and healthy volunteers for each of the six computed biomarkers. **m** Total number of small vessels (with diameter ≤40 µm) in DR layer. **n** Total number of large vessels (diameter >40 µm) in DR layer. **o** Total vessel numbers in DR layer. **p** Total blood volume in DR layer. **q** Thickness of EP layer. **r** Signal density of EP layer as a function of group studied. *P < 0.05, **P < 0.01, and ***P < 0.001, respectively. Scale bar = 500 µm. ns not statistically significant, NDS Neuropathy Disability Score, NSS Neuropathy Symptom Score, EP epidermis, DR dermis

We next investigated the relationship between peripheral diabetic neuropathy and microvasculature via the extracted RSOM label-free biomarkers. Diabetes is a chronic disease with systemic complications that generally evolve with time and affect several systems (e.g., cardiovascular, nervous etc.). For the cardiovascular system in particular, diabetes affects all of parts of the cardiovascular system resulting in cardiovascular disease being the leading cause of death among participants with diabetes. Therefore, the question of understanding severity is perhaps more critical than the separation of diabetic from healthy groups. Indeed, while diabetes encompasses a continuum of stages, a diagnostic test simply separates healthy individuals from participants with diabetes based on a threshold value. Consequently, we foresee RSOM playing an important role in quantifying benchmarks or features associated with these different stages of diabetes. To address this question, we first examined RSOM features obtained from measurements of participants with diabetes without complications and measurements from participants with diabetes and different severities of neuropathy. The severity of diabetic neuropathy was clinically evaluated using the Neuropathy Disability Score (NDS) and the Neuropathy Symptom Score (NSS)^42,43^. We divided the diabetic group into three categories: those with no complications (NC, *n* = 45), those with low score neuropathy (LN, *n* = 13; 1 ≤ NDS ≤ 5 or 1 ≤ NSS ≤ 5) and those with high score neuropathy (HN, *n* = 12; NDS > 5 or NSS > 5). Representative RSOM images from the healthy group and the three diabetic groups are depicted in cross-sectional (sagittal) views (Fig. 3a–d) and coronal views (images parallel to the RSOM scan plane) from the EP (Fig. 3e–h) and DR (Fig. 3i–l) layers. The images confirm a reduced vascular density in the DR with progression of the disease and its complications. Moreover, observation of the coronal views of the EP layer depicts clearly resolved superficial skin ridges in the healthy skin, which change into an amorphous pattern without ridge definition depending on disease status, especially for the groups with diabetic neuropathy.

Quantitative comparisons of the performance of different RSOM features are presented in (Fig. 3f–i). Overall, the count of small vessels (Fig. 3m) demonstrated statistically significant differences between the three diabetic groups, i.e., healthy versus NC (*P* < 0.01), NC versus LN (*P* < 0.05), and LN versus HN (*P* < 0.001). As shown in (Fig. 3m), the neuropathy grade is visible in the count of small vessels (<40 µm diameter). However, the presence of neuropathy had no significant effects on the number of large vessels (Fig. 3n). This finding is also reflected in the total vessel number between the healthy and the NC (*P* < 0.05), as well as between the LN and HN groups (*P* < 0.01). No marked changes were observed between the NC and LN groups. The computation of blood volume (Fig. 3p) demonstrated a similar performance to the small vessel count, with the NC group exhibiting significantly lower blood volume compared to healthy group (*P* < 0.01). The total blood volume was further reduced in the LN compared to NC group (*P* < 0.05). A significant difference in total blood volume was observed between the LN and HN groups (*P* < 0.001).

The EP layers of the NC group were markedly thicker when compared to the healthy volunteers, while neuropathy decreased the epidermal thickness significantly (Fig. 3q). An unpaired *t*-test supported significant differences between the healthy versus NC (*P* < 0.05), as well as the LN versus HN (*P* < 0.05). The differences between the NC versus LN groups were even more significant (*P* < 0.01). The overall optoacoustic signal density of the EP layer (Fig. 3r) decreased in NC groups, compared to the healthy and NC groups (*P* < 0.01).

In addition, we have computed the Spearman correlation values between age/disease duration /HbA1c/BMI with the vascular biomarkers TBV and SVN in the NC, LN, and HN groups, and found no significant correlations as presented in supplementary Tables II and III. We applied multivariate logistic regression analysis to compute the statistics of the two biomarkers TBV and SVN with adjustment of age/disease duration /Hb1Ac /BMI, which showed no significant association between the vascular biomarker and participant parameters (supplementary Table IV).

### Quantification of RSOM biomarkers in diabetic neuropathy and atherosclerosis

**Fig. 4:**
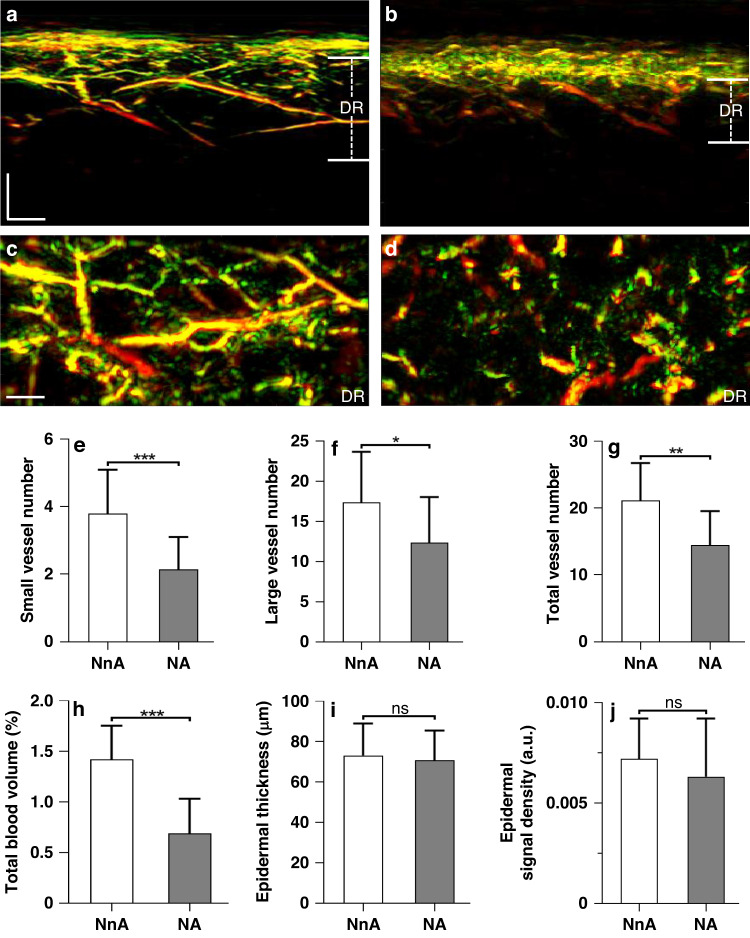
Quantification of RSOM features in participants with diabetic neuropathy and atherosclerosis. Participants with diabetes were grouped as follows: diabetic subjects with neuropathy and no atherosclerosis (NnA, *n* = 25); diabetic subjects with neuropathy and atherosclerosis (NA, *n* = 24). **a** Sectional RSOM image from NnA participant. **b** Sectional RSOM image from NA participant. **c**, **d** MIP images of vascular maps in the coronal views of DR layers corresponding to (**a**, **b**). **e**–**j** Comparisons between the two groups for the following features: **e** total number of small vessels (with diameter ≤40 µm) in DR layer; **f** total number of large vessels (with diameter >40 µm) in DR layer; **g** total number of vessels in DR layer; **h** total blood volume in DR layer; **i** thickness of EP layer; **j** signal density of EP layer. *P < 0.05, **P < 0.01, and ***P < 0.001, respectively. Scale bar = 500 µm. ns not statistically significant. EP epidermis, DR dermis

We were also interested in exploring the association between RSOM features and macrovascular atherosclerosis. Most of the diabetic subjects with atherosclerosis enrolled in this study had also been diagnosed with neuropathy. Therefore, participants with diabetes were divided into two groups: diabetic subjects with neuropathy and no atherosclerosis (NnA, *n* = 25), and diabetic subjects with neuropathy and atherosclerosis (NA, *n* = 24). Representative cross-sectional (sagittal) views and coronal views of the DR layer from the two groups (Fig. 4a–d) showed marked differences. There were significant differences in the numbers of small, large, and the total number of vessels between the group with and the group without atherosclerosis (Fig. 4e–g). The small vessel counts again exhibited the most statistically significant difference (*P* < 0.001) between the two groups, compared with the total vessel count (*P* < 0.01) and large vessel count (*P* < 0.05). In addition, the total blood volume (Fig. 4h) was significantly reduced in diabetic participants with atherosclerosis (*P* < 0.001). Conversely, atherosclerosis had no apparent effect on the epidermal thickness (Fig. 4i) or the optoacoustic signal density of the EP layer (Fig. 4j).

While no other imaging method has previously studied imaging biomarkers in relation to severity of diabetes microangiopathy, the pilot RSOM data collected herein has been shown to be able to classify participants with diabetes based on skin microvascular changes. Typically, previous imaging studies could only detect changes by comparing monitored parameters of healthy and diabetic populations, demonstrating a function that can be also achieved in a straightforward way using a blood test. In comparison, the RSOM features can be used to separate participants with diabetes from healthy individuals with high accuracy. For example, using the small vessel number (SVN) as the differentiating biomarker, a simple classification model demonstrated a 90.2% accuracy with a 93.1% sensitivity, an 80.0% specificity and an area under the Receiver Operator Characteristic (ROC) curve of 0.93 (see Fig. S11). However, we do not envision RSOM for simple diagnostic tests as above, but rather in its possible role to study the state of the vascular system on a personalized basis and, in the future, contribute to assigning a more informative “health score” as elaborated in the discussion.

## Discussion

The skin has been heralded as a window to assessing systemic health conditions. This premise has so far held true for identifying several conditions, for example, manifestations of systemic sclerosis, lupus erythematosus, sarcoidosis, or several types of infections based on superficial features appearing on the upper layers of the epidermis^44^. In this work, we employ RSOM as a novel technique that can provide high-resolution imaging under the skin surface and a detailed assessment of dermal microvasculature and other skin features. RSOM is the only technique available that can non-invasively provide highly detailed three-dimensional images with virtually isotropic resolution and precise cross-sectional images of optical contrast through the deep skin dermis layer. Therefore, it enables new opportunities for three-dimensional investigation of sub-surface skin features and new ways to use the skin in identifying disease than it is possible with the traditional superficial inspection. We note that previous studies based on OCTA have offered in vivo insights into the diabetic skin microvasculature but did not reach the deep skin dermis layer compared to RSOM. Furthermore, in our study, RSOM skin features are associated with the stage of diabetes mellitus, as reflected by the presence of different diabetes-related complications. Successfully accomplishing these goals would introduce a new label-free and portable technology for quantifying diabetes complications, possibly serving in the future as a portable tool for studying and monitoring disease progression with fine precision, complementing symptom-based assessments.

The relationship between skin microvasculature and diabetes stage has not been previously investigated in a non-invasive manner, due to the lack of tools that are capable of detailed assessments of fine vasculature. Likewise, no technologies used in other imaging studies to date have examined the relationship between biomarkers detected in vivo and disease states. High-frequency ultrasound has been applied to assess skin morphology in participants with diabetes mellitus^15–18^. However, speckle effects prevent ultrasound from visualizing microvasculature, i.e., vessels of <100 µm in diameter^45^, without applying contrast agents (microbubbles), which limits routine application in humans. Hyperspectral imaging (HSI) assesses oxy- and deoxy-hemoglobin concentrations in the skin^19,20^, but this method cannot visualize skin microvasculature, as it suffers from low resolution and quantification accuracy caused by variations in skin absorption and scattering properties. Nailfold capillaroscopy circumvents the scattering problem, due to the relative transparency of the nail bed, and has been employed to observe capillary abnormalities of diabetic participants^21,22^. However, thicker, opaque, or pigmented nail folds challenge the accuracy of the measurements^46,47^. OCT has been employed to assess retinal vasculature in relation to diabetic retinopathy^48–51^ or measure the epidermal thickness in participants with type I diabetes mellitus^52^. Similarly, OCT angiography (OCTA) can quantify impairments of the retinal vasculature in participants with diabetic retinopathy compared to the control group^53,54^. In addition, a few studies have applied OCTA to quantify skin microvasculature structure and function, showing the possibility to distinguish between healthy individuals and participants with diabetes^23,24^.

In general, several studies have explored the opportunity to differentiate between healthy volunteers and participants with diabetes based on imaging of the skin^23,24^. Nevertheless, none of these studies thoroughly examined these changes at different stages of the disease, as reported by the presence and severity of relevant complications. Herein, ultra-wideband RSOM resolves skin vessels with diameters ranging from 10 μm to about 150 μm, allowing a detailed understanding of the relationship between disease severity and vessel size, which was not previously possible. The implications of this ability of RSOM are multi-faceted, since successful application of the technology could improve the longitudinal study of diabetes and enable a method to monitor lifestyle or other interventions in a detailed, quantitative way that is not available today. RSOM can play a very different role in comparison to blood glucose measurements. While the latter determines the day-to-day glucose status and is necessary for reducing hypoglycemic incidents, RSOM could monitor an actual state of diabetes using a measure of systemic damage. Importantly, cutaneous microvasculature changes could be non-invasively monitored by means of RSOM frequently, at intervals close together enough that monitoring allows for interventions before the appearance of new clinical symptoms. Regular monitoring would also provide a more precise measure of diabetes progression.

We have previously demonstrated that RSOM can provide detailed images of skin vasculature, and that quantitative information pertaining to dermatological conditions can be extracted from these images^27,32,55,56^. However, it was unclear previously if RSOM would have the sensitivity to capture diabetes-related changes within skin microarchitecture. The ability to capture microvascular changes with RSOM would allow correlations to be drawn with diabetes severity, which had not been examined before. The study herein provided RSOM images of the skin of participants with different diabetic conditions (diabetes severity). Six RSOM label-free biomarkers were extracted from these images: three associated with dermal micro-vasculature (total vessel count, vessel count for vessels <40 μm in diameter, and vessel count for vessels >40 μm in diameter), and three associated with bulk measurements, such as the total blood volume and the thickness and signal density of the epidermis. The precision/accuracy of our segmentation methods was further validated by means of relevant animal studies. More specifically RSOM was also used to image the skin microvasculature in mice. These measurements were finally validated via histological analysis of the same skin region excised after the RSOM measurement. Visual inspection of RSOM images revealed changes in the patterns observed in the different pathologies. Qualitatively, it was generally visible that as diabetes progresses, the vascular density in the dermal layer decreases and the epidermis becomes thinner and less light absorbing. Statistical significance tests performed on features quantitatively extracted from the RSOM images confirmed that all these biomarkers are associated with aspects of diabetes progression and its complications. Moreover, these analyses identified density of vessels <40 μm in diameter to be the most indicative marker of diabetes severity, providing the starkest contrast between the different groups of participants. The identification of small vasculature as the component of the skin that is most affected by diabetes progression highlights the vulnerability of small vessels to systemic effects caused by diabetes and suggests that this marker could possibly be used as a label-free biomarker that indicates diabetes severity.

The results obtained are consistent with findings based on histology studies. Diabetes is known to alter human skin microvasculature, reflecting a systemic effect of the disease. Participants with diabetic neuropathy demonstrate pathological alterations of the microvessels^57^, as also observed by RSOM. Likewise, histological analysis of the skin revealed a 7.2% increase in epidermal thickness in participants with diabetes and without neuropathy and a 16.5% decrease in participants with diabetic neuropathy compared to healthy controls (all *P* < 0.05)^58^, a finding also confirmed in the current study using RSOM (Fig. 3). However, while RSOM offers a comprehensive view of the human skin, histology studies afford only partial observations. For example, previous analyses of thick samples using confocal microscopy^59^ confirmed the presence of decreased vascular densities within the sub-epidermal layers of participants with diabetes compared to healthy individuals. These findings were also consistent with our RSOM readouts (Fig. 3). In contrast, histological analysis of thin samples from superficial skin layers using conventional microscopy^60^ showed that the dermal vascular density was: (i) significantly lower in participants with no or mild neuropathy but (ii) higher in participants with moderate to severe neuropathy, always compared to healthy individuals. RSOM’s ability to capture three-dimensional images of the entire skin in vivo not only enables longitudinal studies on the same individual, but also more complete analyses of the effects of diabetes on the skin.

RSOM represents a potential paradigm shift in the non-invasive evaluation of skin vasculature, well beyond the current state-of-the-art. Depending on the wavelength employed, the method can penetrate several millimeters under the skin surface. Using laser with a wavelength of 532 nm in this study, we focused on visualizing the first millimeter of the skin while retaining high contrast due to the relatively high absorption of hemoglobin in the green region. Highly detailed RSOM images were showcased herein both as cross-sectional images and as coronal images from different layers. No other method today can achieve this imaging detail and depth, using label-free operation. Moreover, an RSOM system is cost-efficient and can be made highly portable to allow disseminated use. Therefore, the results point to the use of RSOM as a highly potent strategy for offering a quantitative assessment of the effects of diabetes on skin and possibly in diabetes staging. Other tests that can measure skin changes include simple visual assessment of the skin surface or Doppler imaging^61^ to assess changes in blood flow in the skin due to stimuli, such as the post-occlusive increase of shear stress, hyperthermia, or drug applications^62,63^. Optical coherence tomography also offers partial views of skin microvasculature^33,64^. However, none of these methods offers the quality and detail of RSOM, and consequently, none of these methods have been considered for assessing skin microvasculature and diabetes-related alterations.

There are several reports that microvascular changes occur early in the course of diabetes^3,5,7,8,59,60^. This observation points to a prospective study in high-risk populations to examine RSOM biomarkers at different stages of disease development, from pre-diabetes to diabetes. Such a study could further expand the possible applications of RSOM, not only as a tool to stage and monitor the progression of diabetes, but also as a means for early detection. For example, with RSOM being safe, portable, and non-invasive, such measurements could be readily extended to larger-scale and multi-center studies, in particular as it concerns collection of data for identifying the early detection power of the RSOM biomarkers. Likewise, optimization studies could be performed to identify potential differences in different skin loci of RSOM acquisition and select locations that further improve performance. Our current RSOM system is equipped with a monochromatic laser and provides access to total hemoglobin measurements but does not differentiate further between oxygenated and deoxygenated hemoglobin. In the future, we aim to employ multispectral RSOM imaging (see Fig. S12) to quantify changes in both oxygenated and deoxygenated hemoglobin and extract information on skin metabolism and oxygenation. These measurements can be used to explain the pathophysiology of chronic wounds and prognose disrupted wound healing processes in participants with diabetes mellitus that result in an enormous cost burden and decreased of quality of life. Another potential use of RSOM could be in improving classification of participants with diabetes by assigning a quantitative “health score” based on the status of skin features. The current practice of separating diabetic and healthy populations based on a simple threshold is a sub-optimal strategy for prevention and diabetes healthcare^25,26^ that converts a gradual progression to a binary distribution. It has been noted^25,26^ that it is possible to administer better healthcare when the population is not separated by a threshold but rather assigned a score so that individuals are better alerted to their condition, monitored closely and be considered for a prevention program. A non-invasive portable and label-free technology such as RSOM could play a vital role in offering quantitative metrics in high-risk populations and evaluating possible interventions. Although such strategies apply primarily to participants with type 2 diabetes, the overall need to improve diabetes staging has been outlined for both types of diabetes 1 and 2^25,26^.

In summary, we presented the first imaging study correlating in vivo biomarkers to diabetes severity. The data also represents the first optoacoustic mesoscopy images of the skin of participants with diabetes, as well as the first non-invasive in vivo study of the effects of diabetes and its complications on skin microvasculature and skin microanatomy in participants with diabetes mellitus. RSOM extracted six label-free biomarkers associated with skin morphology and microvasculature and identified fine vasculature as the feature most sensitive to progress of complications of diabetes. This finding further shows the promise of RSOM as a potential point-of-care device for quantifying systemic complications of diabetes and providing a quantitative score indicative of disease stage. Due to its safety, portability, low cost, high image quality, and ability to quantify label-free biomarkers, RSOM may offer a paradigm shift in the clinical characterization of diabetes, assessment of interventions and in prevention programs.

## Materials and methods

### RSOM imaging system

We employed an in-house portable RSOM imaging system featuring a transducer with a 10–120 MHz bandwidth and central frequency of ~50 MHz (Fig. 1a), which has been described in detail elsewhere^27,65^. Illumination was provided by a pulsed laser at a wavelength of 532 nm. The repetition rate of the laser was 1 kHz, yielding an optical fluence of 3.75 µJ/mm^2^, which is far below the safety limit according to the American National Standards for Safe Use of Lasers in humans^66^. An optically and acoustically transparent plastic membrane was affixed on the participant’s skin over the examined position. Both the laser output and ultrasound transducer (UT) were mounted on the same scanning head placed close to the membrane to position the focal point of the ultrasound detector slightly above the skin surface and maximize detection sensitivity. For every measurement, we conducted a calibration step by placing the RSOM transducer in a position so that the most superficial skin signal could be clearly detected. The scanning head contained water as a coupling medium. Two mechanical stages (PI, Germany) were used to move the RSOM head. Both the laser and the controller of the mechanical stages were placed inside a plastic case, which ensured laser safety for all participants, as shown in Fig. S1. The scanning field of view is 4 × 2 mm^2^ with a step size 7.5 µm in the fast axis and 15 µm in the slow axis. The axial and lateral resolutions of RSOM is about 4.5 μm and 18.4 μm respectively, our previous characterization measurements showed that the resolutions generally remained constant throughout the whole dermis (1.5 mm deep)^27,65^.

### Recruitment, data grouping, and statistical analysis

One hundred and two (*n* = 102) participants with diabetes and 48 age-matched healthy volunteers were scanned in total. Participants with diabetes and healthy volunteers were recruited following approval from the Ethics Committee of the Faculty of Medicine of the Technical University of Munich (Protocol No 109/17S). All participants with and without diabetes gave written informed consent before the planned RSOM examination. RSOM data quality was evaluated based on our previously developed RSOM quality evaluation approach and low-quality data was excluded^67^. Significantly, higher melanin concentrations in skin could decrease the penetration depth of our imaging system. The scanned regions from participants with strong melanin were excluded to minimize the influence of the melanin. Finally, RSOM data from 95 participants with diabetes and 48 healthy volunteers were included in the current analysis. Included participants were split into three main groups based on the presence of relevant complications, such as peripheral neuropathy and macrovascular atherosclerosis and peripheral artery disease (ASCVD/PAD). Group A consisted of 45 participants with diabetes but without complications (neither neuropathy nor ASCVD/PAD). Group B included 25 participants with diabetic neuropathy but without ASCVD/PAD. Group C consisted of 25 participants with diabetic neuropathy and ASCVD/PAD. The presence and severity of peripheral neuropathy was assessed by using the neuropathy symptom score (NSS)^42^ and painful symptoms were quantified with a visual analog score ranging between 1 and 10. Peripheral neuropathy was also assessed using the neuropathy disability score (NDS)^43^ in the range of 1 to 10, which included 10 g mono-filament testing, tuning fork vibration perception, pin prick perception, and temperature perception.

To quantify the effects of neuropathy, Group B was further divided into two sub-groups with the low NDS and NSS scores (*n* = 13, 1 ≤ NDS ≤ 5 or 1 ≤ NSS ≤ 5) and high scores (*n* = 12, NDS > 5 or NSS > 5). Participants with ASCVD/PAD were characterized either by a history of cardiovascular events or by having undergone an arterial revascularization procedure. PAD was characterized by the presence of a clinically relevant stenosis in the peripheral arterial system, as diagnosed by Doppler ultrasound measurements, which was associated with intermittent claudication.

### Participant preparation and image acquisition

Participants were asked to consume no caffeine or food for at least 4 h before the RSOM measurements. They were placed in a quiet and dark room and left to relax for at least 5 min. The temperature of the room was held stable at 23 °C during the whole procedure. The measurements were performed with the participants in the supine position. Each participant was scanned at 2 symmetric regions of interest (ROIs, 4 × 2 mm^2^) over the pretibial region of the distal lower limb. The scan of the dominant leg was used for further analysis. The pretibial region was used as representative of skin microcirculation since the participants with diabetes are prone to developing cutaneous alterations at this very position. Each RSOM scan lasted ~70 s. Before each scan, the skin was cleaned with alcohol wipes. Both the participants and the operators used appropriate goggles for laser safety reasons.

### Image reconstruction

Acquired RSOM signals were divided into two frequency bands, 10–40 MHz (low) and 40–120 MHz (high), for the 10–120 MHz bandwidth. Signals in the two different bands were independently reconstructed. Reconstructions were based on beam-forming algorithms that generated three-dimensional images^65^. The reconstruction algorithm was accelerated by parallel computing on a graphics processing unit and improved by incorporating the spatial sensitivity field of the detector as a weighting matrix. The reconstruction time of one bandwidth takes about 5 min with voxel size of the reconstruction grid at 12 µm × 12 µm × 3 µm. The two reconstructed images ${R}_{{low}}$ and ${R}_{{high}}$ corresponded to the low- and high frequency bands. A composite image was constructed by fusing ${R}_{{low}}$ into the red channel and ${R}_{{high}}$ into the green channel of an RGB image. A weighting factor was introduced for modulating the intensity of the high-frequency band image. The detail process has been introduced in our previous work^27^. The RSOM images can be rendered by taking the maximum intensity projections of the reconstructed images along the slow axis or the depth direction as shown in Fig. 2.

### Data quality control

For this study, we recruited 103 participants with diabetes and 48 healthy volunteers and recorded 302 RSOM measurements (two scans per person). Our previous studies have shown that motion can significantly affect image quality, although our motion correction algorithms can offer marked improvements^67,68^. However, various motions from physiological displacements due to arterial pulsation and heartbeat and unintentional movements of the participant may lead to inconsistent motion correction improvements. Therefore, we developed a quality control scheme based on the amount of motion in the raw data that classifies the quality of data collected. The quality control scheme enables the selection of high-quality datasets, in which the motion is minimal enough for the motion-correction algorithm to correct, resulting in consistent correction improvements and uniform image quality for quantitative analysis^68^. After the data quality evaluation, RSOM datasets of 8 participants with diabetes were excluded due to serious motion and low image quality.

### Skin layer and microvasculature segmentation and calculation of RSOM-based biomarkers

For layer segmentation, RSOM images were first flattened based on our surface detection approach^68^. The reconstructed volume of the selected frequency band (10–40 MHz) was split into four stacks with 0.5 mm thickness along the slow scanning axis. Then, the epidermis layer in the MIP image of each stack was automatically segmented by a graph theory and dynamic programming-based approach (see Fig. S2)^40^. The segmented boundaries of the epidermis layer from the four stacks were smoothed to achieve the final segmented results, as shown in (Figs. 1c and S2). The thickness of the epidermis layer was calculated as the average width of the four segmented boundaries. Additionally, the signal density of the epidermis layer was determined as the ratio between the sum of the pixel intensity in the epidermis layer and the total segmented volume of the epidermis layer in the 4 × 2 mm^2^ scanning region.

The dermis layer was segmented starting from the bottom boundary of the epidermis layer and extending 1.5 mm deep. In the 4 × 2 mm^2^ scanning region, the total blood volume in the segmented dermis layer was calculated as ratio $\left(\frac{N}{{\rm{T}}}\right)* 100$, where *N* represents the number of voxels with intensities above 20% of the maximum voxel intensity, and $T$ is the total voxel number inside the 4 × 2 × 1.5 mm^3^ volume. Afterwards, the vascular mask in the dermis was segmented by the multi-scale matched filter-based vessel segmentation algorithm, as shown in (Fig. 1d)^41^. Based on the mask, vessel boundaries were extracted, and the corresponding width of the boundaries was calculated as the vessel diameter (Figs. 1d and Fig. S3). Then, the centerlines of vessel boundaries were extracted, and junction points of the centerlines were counted as the total vessel number (see Fig. S3). To reduce noise or artifacts, we removed isolated vessels with lengths <5 pixels (20 µm spatial resolution divided by 3 µm pixel size is ~7). The total vessel number was further divided into the small vessel group and the large vessel group, which can be used to investigate the diabetes effects on different size of vessels. The small vessel number was determined based on the number of junction points, where the average diameter of the connected vessel was <40 µm. Correspondingly, the large vessel number was computed as the number of junction points, where the average diameter of the connected vessel was >40 µm. The 40 µm cut-off is a popular value for differentiating small arterioles and venules from larger or even smaller (5–10 μm) capillaries^69^. Furthermore, smaller vessels (<40 μm) reside only within the epineurium and endoneurium^70^, and endoneurial microvessels are affected with the diabetic nerves resulting in impaired blood supply (vasa nervorum) and thus diabetic neuropathy^71^. We also analyzed this cut-off value of 40 μm based on vessel diameter distributions of 20 healthy volunteers and 20 participants with diabetes as shown in Fig. S5. The vessel diameter was equally categorized into 10 groups according to size (10 µm size ranges from 10 µm in diameter to 100 µm or more than 100 µm to investigate the effects of diabetes on different vascular beds. We noticed that more significant differences of vessel diameter were found above the cut-off value of 40 μm when comparing healthy participants against participants with diabetes. Depending on the vessel characteristics of the studied participants with and without diabetes, the cut-off value can be altered to maximize sensitivity or specificity for the intended application. In addition, the relation between RSOM features and age (see Fig. S6), disease duration (see Fig. S7), body mass index (BMI) (see Fig. S8), and HbA1c values (see Fig. S9) were investigated and shown in the supplementary results. We found that these parameters did not show obvious correlation with the RSOM biomarkers and thus they did not significantly influence the outcome of our study.

### Validation of the layer and vessel segmentation methods

The accuracy of the RSOM biomarker computation was determined by the layer and vessel segmentation methods. In RSOM images, the epidermal layer signal mostly derives from the melanin generating a low frequency layer structure, while the microvasculature contains higher frequency contents. It is very easy to separate visually the epidermal layer from the dermal vasculature in RSOM images. To validate the layer segmentation method, we compared the results of the proposed automatic segmentation method with the manual segmentation performed by two well-trained and independent observers as shown in Fig. S2. The correlation coefficients between the automatic and manual segmentation methods are 0.92 (Observer 1) and 0.96 (Observer 2). Furthermore, we collected RSOM data from the skin of the hip area (4 × 2 mm^2^) of 8 healthy mice and compared with the corresponding histological images. The animal measurements were performed in full compliance with the institutional guidelines of the Helmholtz Center Munich and with approval from the Government District of Upper Bavaria. All scanning parameters of the mouse measurements followed the same configurations of the human measurements. The RSOM datasets of the 8 mice were reconstructed and analyzed following the same analysis procedure of the human data. The RSOM image of mouse skin was segmented into dermis and hypodermis layers, and the vasculature in the hypodermis layer was further segmented to compute the RSOM biomarker (total blood volume). In addition, the dermis thickness of each mouse was calculated in both histological and corresponding RSOM images. CD31 immunostaining was performed to evaluate vessel footprints. The total blood volume in the histological image was computed as the ratio between the vessel marker area and the total hypodermis area, while the total blood volume of RSOM mouse image was computed using the same analysis method of the participants with diabetes data. As shown in Fig. S4, the dermis thicknesses and total blood volumes showed very good correlations between values obtained from the histological and RSOM images (the correlation coefficients values are 0.94 and 0.91 respectively). Furthermore, our previous work has made a comparison of capillary imaging by conventional nailfold capillaroscopy and RSOM^55^. The capillary diameter and capillary density, which computed from the segmented RSOM images, were correlated well with the nailfold capillaroscopy. Moreover, our approaches to segment RSOM skin features (including the epidermis layer thickness and dermal vasculature) were validated in previous work using histology^27^.

### Statistics

A total number of 95 participants with diabetes and 48 healthy volunteers were grouped together to compute biomarkers. All metrics were displayed into column table with mean value and standard deviations as error bar. Information of age/disease duration /Hb1Ac /BMI between healthy controls and participants with diabetes, and differences between the groups with diabetes and subgroups or individuals with Type 1 or Type 2 diabetes were evaluated by the mean of the Mann–Whitney U-test as shown in Table 1 and supplementary Table I to Table V. To assess the significance of the statistical differences for the metrics between healthy and diabetic groups, and sub-groups among participants with diabetes, we performed parametric tests (unpaired *t* test) for normally distributed data; otherwise, nonparametric tests (Mann–Whitney *U* test) were applied. We have computed the Spearman correlation to show the relationship among age/disease duration /HbA1c /BMI with the vascular biomarkers TBV (Total blood volume) and SVN (Small vessel number). In addition, we also applied multivariate logistic regression analysis to compute the statistics of the two biomarkers TBV and SVN between different diabetic groups with adjustment of age/disease duration /Hb1Ac /BMI, and in the analysis of the diabetic neuropathy groups as well. For the multiple regression analysis, all variables that were significantly associated with RSOM biomarkers in the univariate analysis were included in the model. Statistical significance was defined at *P* < 0.05.

### Supplementary information

Supplementary information

Supplementary Movie 1

Supplementary Movie 2
