# Supplementary material for: Opening a window to skin biomarkers for diabetes stage with optoacoustic mesoscopy
Source: Light Sci Appl. 2023 Sep 18;12:231. doi: 10.1038/s41377-023-01275-3 (PMC10505608; doi:10.1038/s41377-023-01275-3)
Supplement: Supplementary file 1 — Supplementary information [file 41377_2023_1275_MOESM1_ESM.docx]

**Supplementary Information for**

**Opening a window to skin biomarkers for diabetes stage with optoacoustic mesoscopy**


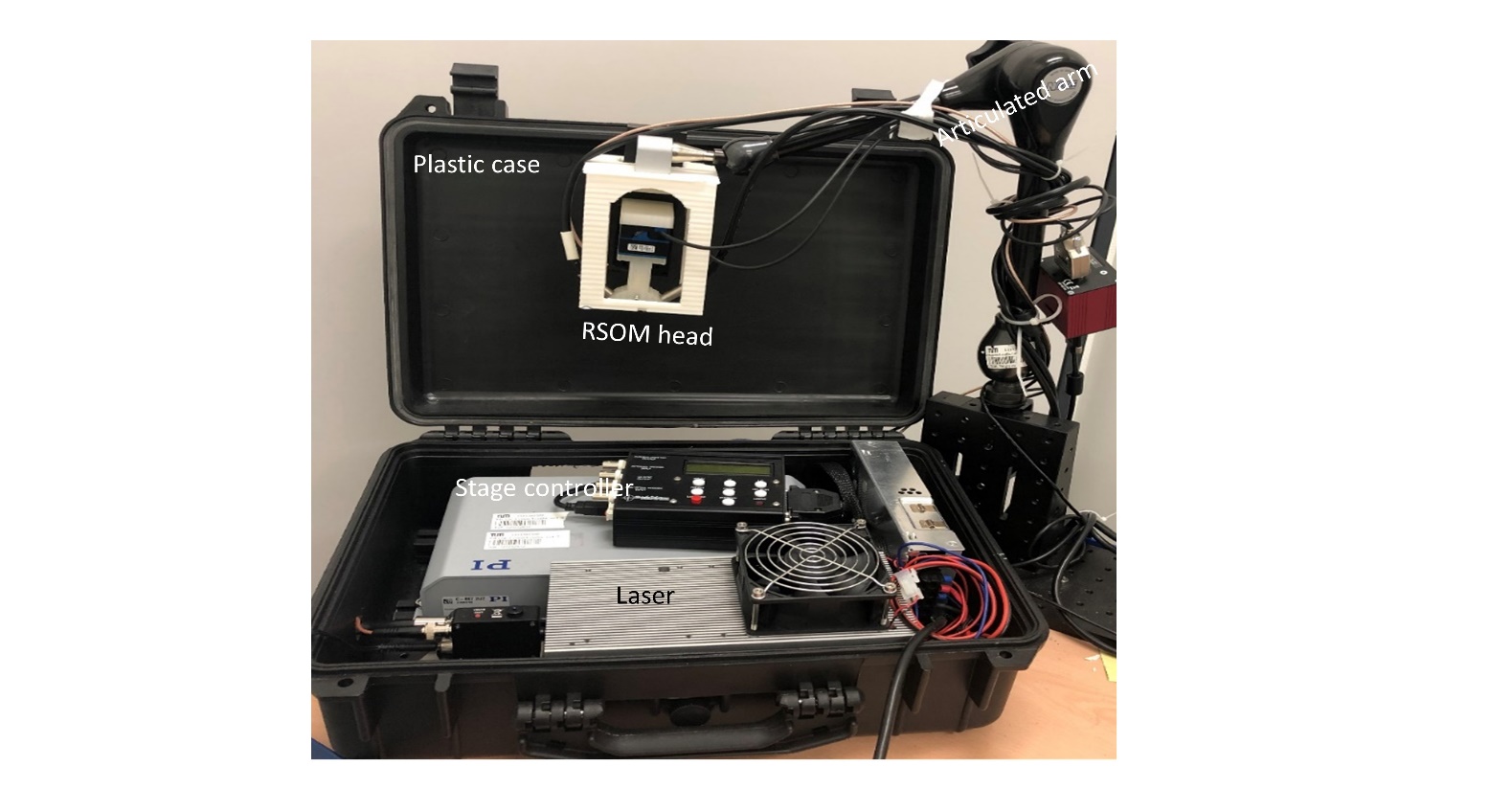


Figure S1. **Picture of the portable RSOM imaging system**. The laser source and the stage controller are contained within a plastic suitcase, creating a controlled laser safety environment during the measurement of the participants with or without diabetes mellitus.

**Layer segmentation method and validation**. To segment the epidermis (EP) layer, RSOM images were first flattened based on our surface detection approach^1^. The reconstructed volume of the selected frequency band [10-40 MHz, Fig. S2a] was split into four stacks with 0.5 mm thickness along the slow scanning axis. Then, the epidermis layer in the MIP image of each stack was automatically segmented by a graph theory and dynamic programming-based approach^2^. To validate the layer segmentation method, we randomly selected 30 RSOM images (from the healthy volunteers and participants with diabetes) and compared the results of the automatic segmentation method with the manual segmentation performed by two well-trained and independent observers as shown in Fig. S2f. The correlation coefficients between the automatic and manual segmentation methods are 0.92 (Observer 1) and 0.96 (Observer 2).


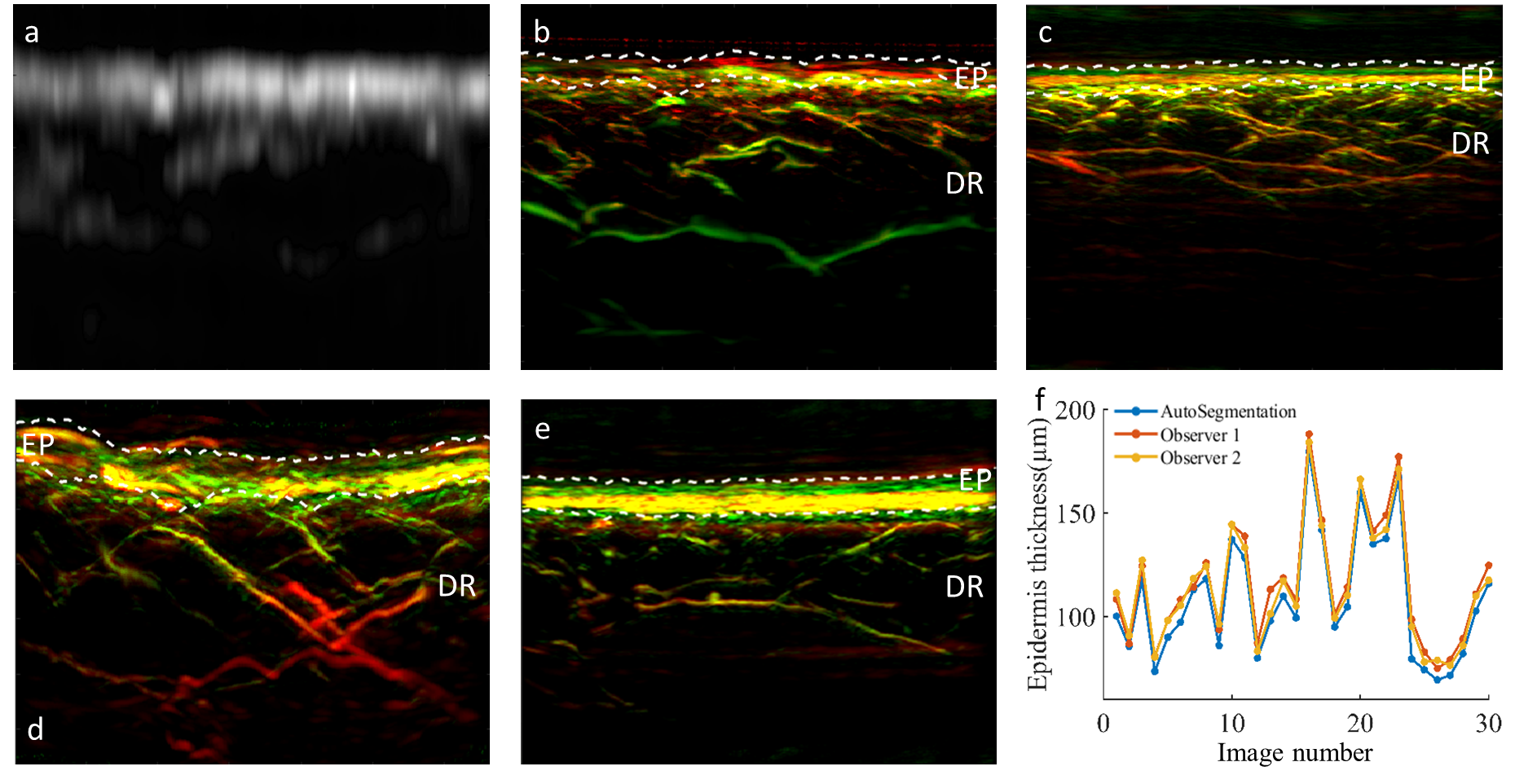


Figure S2. **RSOM image layer segmentation**. (a) The reconstructed RSOM image of low frequency (10-40 MHz) signals, which mainly contain the epidermis layer structure originated from the skin pigment melanin. (b) The corresponding reconstructed RSOM image of (a) in the full frequency bandwidth, where the white dashed lines segment the epidermis layer. (c-e) Validation results of the layer segmentation on RSOM images of several healthy volunteers and participants with diabetes. (f) Comparisons of the layer segmentation results of 30 RSOM images between the automatic and manual segmentation methods by two observers based on human vision showing good correlations. EP: Epidermis, DR: Dermis.


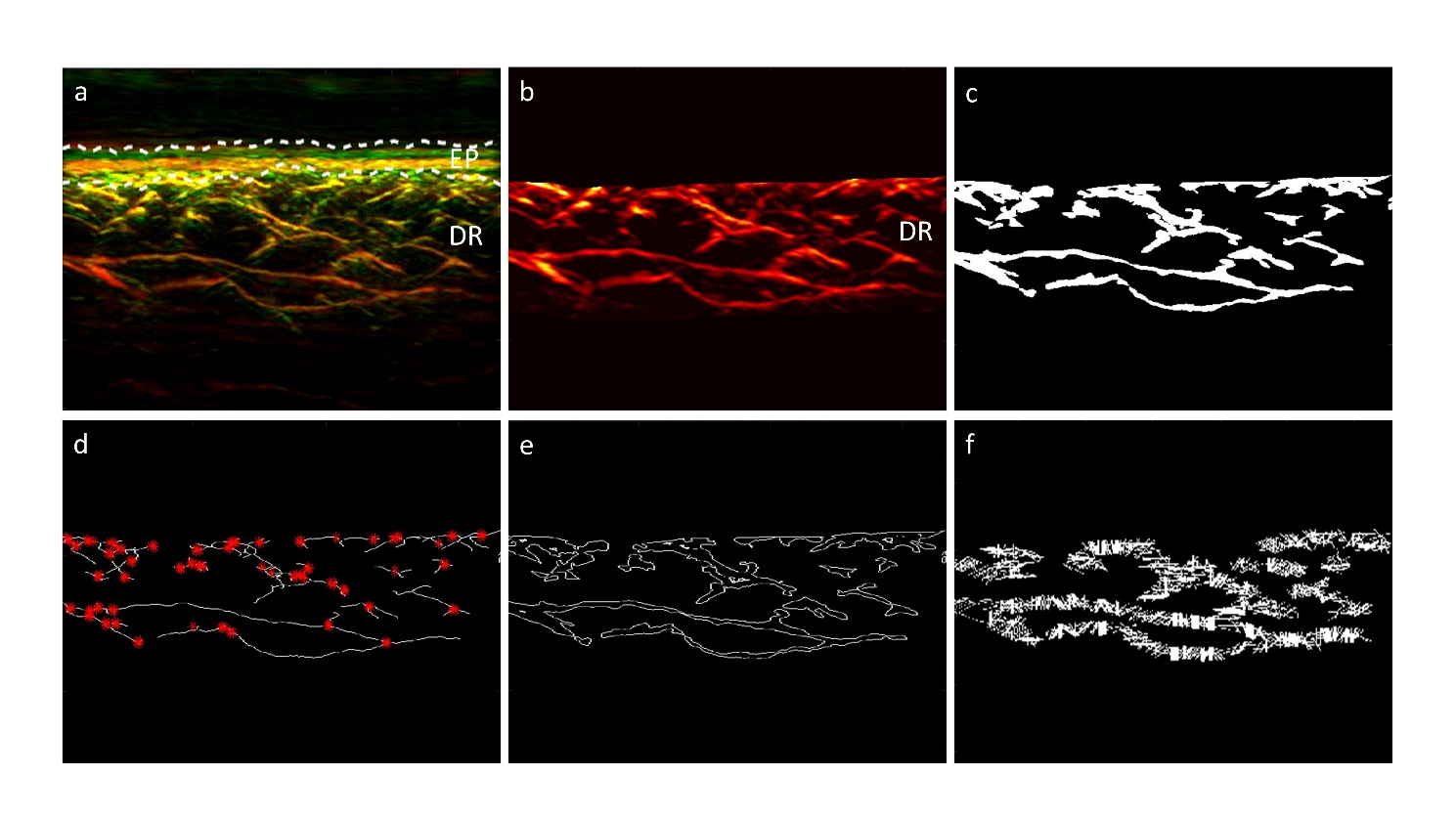


Figure S3. **Automatic vessel segmentation of the dermal vasculature and computation of biomarkers**. (a) Cross-sectional RSOM image from a healthy volunteer. The white dashed lines segment the epidermal layer and dermis layer; (b) The segmented dermal layer; (c) The binary image of the segmented dermal vasculature; (d) The centerlines of the segmented binary image (c); Red dots mark the branches of vessels, used to count the vessel number; (e) The segmented boundaries of the binary image (c); (f) Computation of the diameter of the segmented vessel boundaries (e). EP: Epidermis, DR: Dermis.

**Validations of the layer and vessel segmentation method**. To validate the RSOM computed biomarkers, we collected RSOM data from the skin of the hip area (4×2 mm^2^) of 8 healthy mice and compared with the corresponding histological images. As shown in Fig. S4a and S4c, the RSOM image of the mouse skin was segmented into dermis and hypodermis layers, and the vasculature in the hypodermis layer was further segmented to compute the total blood volume. In addition, the dermis thickness of each mouse was calculated in both histological (Fig. S4c) and corresponding RSOM images (Fig. S4a). CD31 immunostaining was performed to evaluate vessel footprints (black arrows in Fig. S4c). The total blood volume in the histological image was computed as the ratio between the vessel marker area and the total hypodermis area, while the RSOM biomarker (total blood volume) was computed using the same analysis method of the human data. The dermis thickness and total blood volume showed very good correlations between the histological and RSOM images (the correlation coefficient values are 0.94 and 0.91, respectively) shown in Fig. S4d.


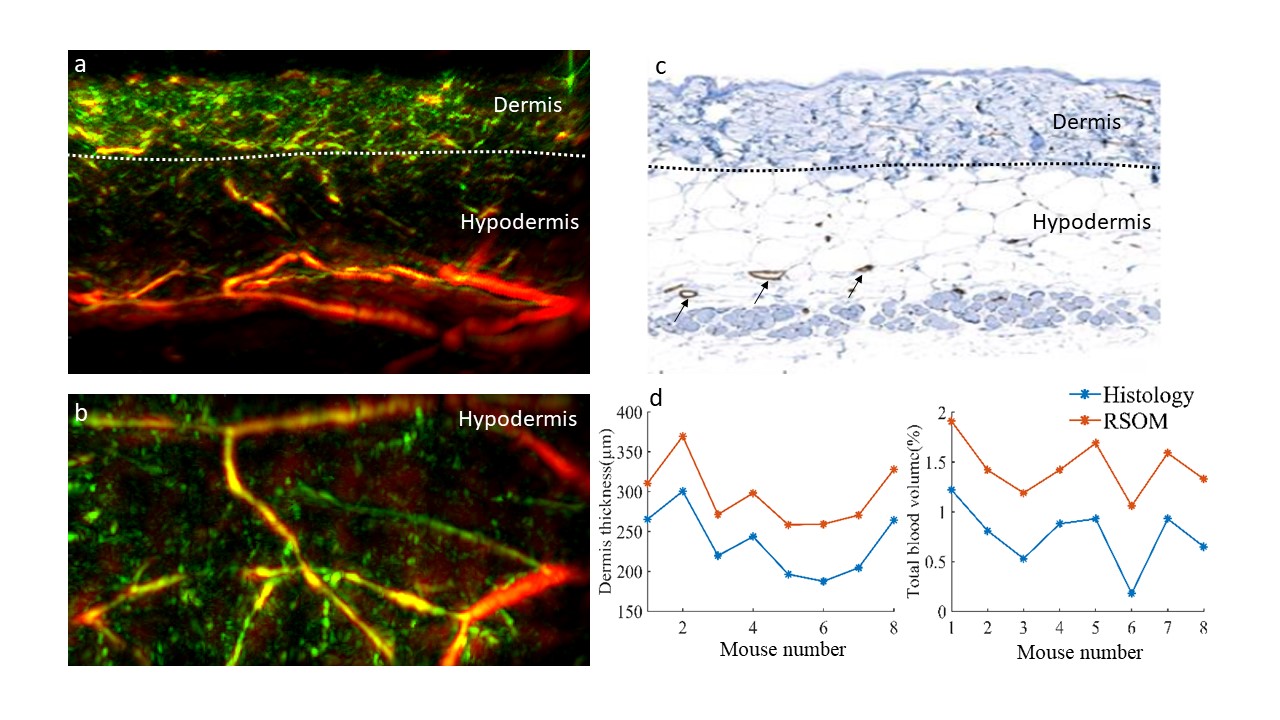


Figure S4. **Validations of layer and vessel segmentation methods on animal data with histology**. (a) Cross-sectional RSOM image of the skin obtained from the hip area of ​​a healthy male mouse. The dermis and hypodermis layers of mouse skin were automatically segmented as indicated by the white dashed line. (b) Corresponding MIP RSOM image of the microvasculature in the coronal view of the hypodermis layer. (c) CD31 immunostaining of the scanned mouse skin, where the black dashed line separates the dermis and hypodermis, in good correlation with the RSOM image (a). The vessels are indicated by the black arrows. To validate the segmentation method, the results of automatic segmentation of RSOM images were compared against manual segmentation of histological images using 8 mice of different weights. (d) The dermis thicknesses and total blood volumes were calculated from histological images and RSOM images, with high correlations found between values obtained from RSOM and histological images.

**Selection of the cut-off vessel diameter to distinguish between participants with and without diabetes.** In this study, vessels were categorized according to size (10 µm size ranges from 10 µm in diameter to 100 µm or more than 100 µm) to investigate the effects of diabetes on different vascular beds. The cut-off value was determined based on vessel diameter distributions of 20 healthy volunteers and 20 participants with diabetes as shown in Fig. S5. We noticed that more significant differences were found above the cut-off value of 40 µm when comparing healthy participants against participants with diabetes. Depending on the vessel characteristics of the study participants with and without diabetes, the cut-off value can be altered to maximize sensitivity or specificity for the intended application.


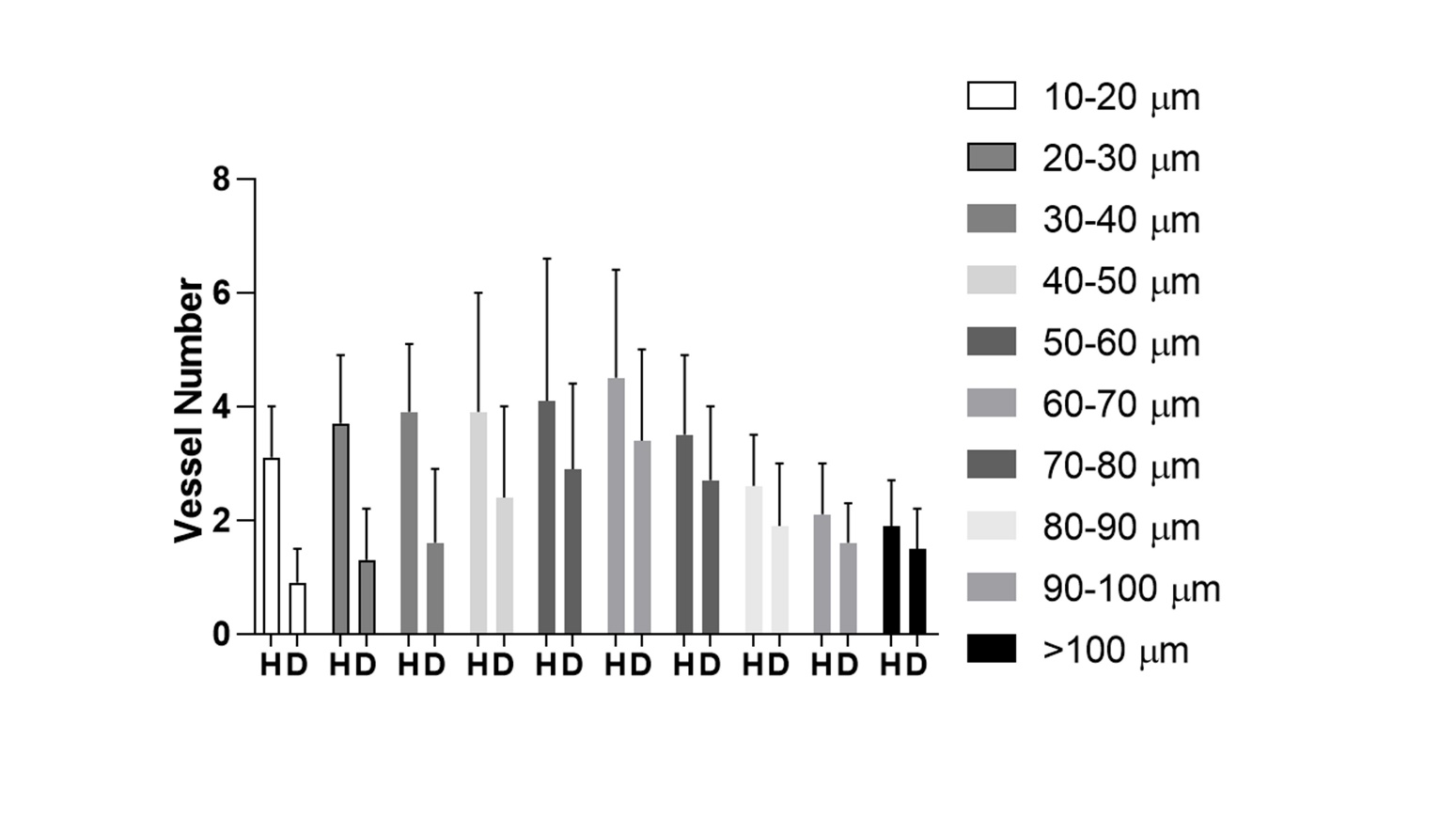


Figure S5. **Vessel number distributions in healthy volunteers and participants with diabetes**. All vessels of 20 healthy (H) volunteers and 20 participants with diabetes (D) are categorized into 10 groups based on the vessel diameter. Values are means with standard deviations shown as error bars.

To understand the effects of diabetes on the vascular biomarker, we computed the Spearman correlation value between age/disease duration/HbA1c/BMI with the vascular biomarker TBV and found no significant correlations as shown in supplementary Table I. We further applied multivariate logistic regression analysis to estimate the significance of TBV between the healthy and diabetic groups including variables of age and BMI, which showed no significant association between the vascular biomarker and patient parameters.

**Table I** Correlation and multivariate logistic regression analysis between vascular biomarker (Total blood volume: TBV) and patient parameters.

|  | Correlation^*^ | |  | Multivariate^**^ | |
| --- | --- | --- | --- | --- | --- |
|  | *r* | *p* |  | *β* | *p*  0.0007  n/a  n/a  0.0004 |
| Age (years) | -0.08 | 0.54 |  | 0.92 |  |
| Disease duration (years) | -0.04 | 0.69 |  | n/a |  |
| HbA1c (%) | -0.014 | 0.90 |  | n/a |  |
| BMI (kg/m²) | -0.13 | 0.42 |  | 0.83 |  |
| Note: *The Spearman correlation value was calculated between TBV and the various patient parameters and no significant correlations were found; **Multivariate logistic regression analysis was applied to compute the significance of vascular biomarker TBV between the healthy and participants with diabetes groups including the variables of age and BMI, which still show significant differences between the healthy and patient groups. n/a: Not applicable. BMI: Body mass index, HbA1c: Glycated hemoglobin. | | | | | |

For participants with neuropathy, we carefully compared age, disease duration, HbA1c and BMI between NC (participants with diabetes and no complications), LN [participants with diabetes and low score neuropathy (1≤ NDS ≤ 5 or 1≤NSS ≤ 5)] and HN [participants with diabetes and high score neuropathy (NDS > 5 or NSS > 5)] as shown in supplementary Table II. There are no significant differences in age, HbA1c and BMI values among the three groups and only the duration of disease between the NC and LN differs significantly. We also computed the Spearman correlation between age/ duration of disease /HbA1c/BMI with the vascular biomarker TBV of the three groups and we found no significant correlation as shown in supplementary Table III.

**Table II** Participant information for diabetic neuropathy analysis.

|  | NC (n=45) | LN (n=13) | P value^*^ | HN (n=12) | P value^**^ |
| --- | --- | --- | --- | --- | --- |
| Age (years) | 63.1±18.7 | 68.8±8.3 | ns | 71.3±10.8 | ns |
| Disease duration  (years) | 12.1±11.2 | 28.7±18.9 | <0.05 | 27.1±14.2 | ns |
| HbA1c (%) | 7.1±1.6 | 7.2±0.7 | ns | 7.3±0.7 | ns |
| BMI (kg/m²) | 27.4±7.6 | 30.6±10.7 | ns | 29.8±6.0 | ns |

Note: * NC group versus LN group; **LN group versus HN group [participants with diabetes and high score neuropathy (NDS > 5 or NSS > 5). NC: participants with diabetes and no complications, LN: participants with diabetes and low score neuropathy (1≤ NDS ≤ 5 or 1≤NSS ≤ 5) and HN: participants with diabetes and high score neuropathy (NDS > 5 or NSS > 5). We performed parametric tests (unpaired t-test) for normally distributed data; otherwise, non-parametric tests (Mann Whitney U test) were applied. Statistical significance was assumed at P < 0.05. ns: Not statistically significant. BMI: Body mass index, HbA1c: Glycated hemoglobin.

**Table III** Correlation between vascular biomarker (Total blood volume: TBV) and participant parameters in the diabetic neuropathy groups.

| Variable | NC | | LN | | HN | |
| --- | --- | --- | --- | --- | --- | --- |
|  | *r* | *p* | *r* | *p* | *r* | *p* |
| Age (years) | -0.08 | 0.41 | -0.11 | 0.41 | -0.18 | 0.42 |
| Disease duration (years) | -0.04 | 0.41 | -0.17 | 0.27 | -0.09 | 0.31 |
| HbA1c (%) | 0.019 | 0.43 | -0.13 | 0.34 | -0.24 | 0.29 |
| BMI (kg/m²) | -0.19 | 0.57 | -0.14 | 0.41 | -0.07 | 0.38 |
| Note: The Spearman correlation value was computed between TBV and the patient parameters in each group with no significant correlations found. NC: participants with diabetes and no complications, LN: participants with diabetes and low score neuropathy (1≤ NDS ≤ 5 or 1≤NSS ≤ 5) and HN: participants with diabetes and high score neuropathy (NDS > 5 or NSS > 5). BMI: Body mass index, HbA1c: Glycated hemoglobin. | | | | | | |

To examine the effects of age, disease duration, HbA1c and BM, as shown in supplementary Table IV, we applied multivariate logistic regression analysis including the variables of age, duration of the disease, HbA1c and BMI, which did not show significant effects in the statistical analysis among the three groups NC, LN, and HN.

**Table IV** Multivariate logistic regression analysis of participant groups with neuropathy, including patient parameters.

| Patient parameters | Patient groups | *P value*^*^ | *P value with adjustment*^**^ |
| --- | --- | --- | --- |
| Age (years) | NC and LN | <0.05 | <0.05 |
|  | LN and HN | <0.001 | <0.001 |
| Disease duration  (years) | NC and LN | <0.05 | <0.05 |
|  | LN and HN | <0.001 | <0.001 |
| HbA1c (%) | NC and LN | <0.05 | <0.05 |
|  | LN and HN | <0.001 | <0.001 |
| BMI (kg/m²) | NC and LN | <0.05 | <0.05 |
|  | LN and HN | <0.001 | <0.001 |

Note: * We performed parametric tests (unpaired t-test) for normally distributed data; otherwise, nonparametric tests (Mann Whitney U test) were applied. Values of P < 0.05 were considered statistically significant. **Multivariate logistic regression analysis was applied to compute the significance of the vascular biomarker TBV between the patient groups NC and LN, and LN and HN including the variables of age, disease duration, Hb1Ac, and BMI, which did not affect the statistical results. NC: participants with diabetes and no complications, LN: participants with diabetes and low score neuropathy (1≤ NDS ≤ 5 or 1≤NSS ≤ 5) and HN: participants with diabetes and high score neuropathy (NDS > 5 or NSS > 5). BMI: Body mass index, HbA1c: Glycated hemoglobin.

We compared the characteristics of participants with type 1 and type 2 diabetes, as shown in supplementary Table V. We found that there were no significant differences in age, Hb1Ac and BMI values between the two groups and that only the type 1 group had significantly longer disease duration compared with the type 2 group.

**Table V** Characteristics of participants with type 1 and type 2 diabetes

|  | Type 1 (n=21) | Type 2 (n=74) | P value |
| --- | --- | --- | --- |
| Age (years) | 67.2±12.4 | 68.2±11.4 | ns |
| Disease duration (years) | 32.0±19.3 | 16.1±11.5 | <0.05 |
| Hb1Ac (%) | 7.5±0.8 | 7.0±1.2 | ns |
| BMI (kg/m²) | 27.1±3.4 | 26.9±8.1 | ns |

Note: We performed parametric tests (unpaired t-test) for normally distributed data; otherwise, nonparametric tests (Mann Whitney U test) were applied. Statistical significance was assumed at P < 0.05, ns: Not statistically significant. BMI: Body mass index, HbA1c: Glycated hemoglobin.


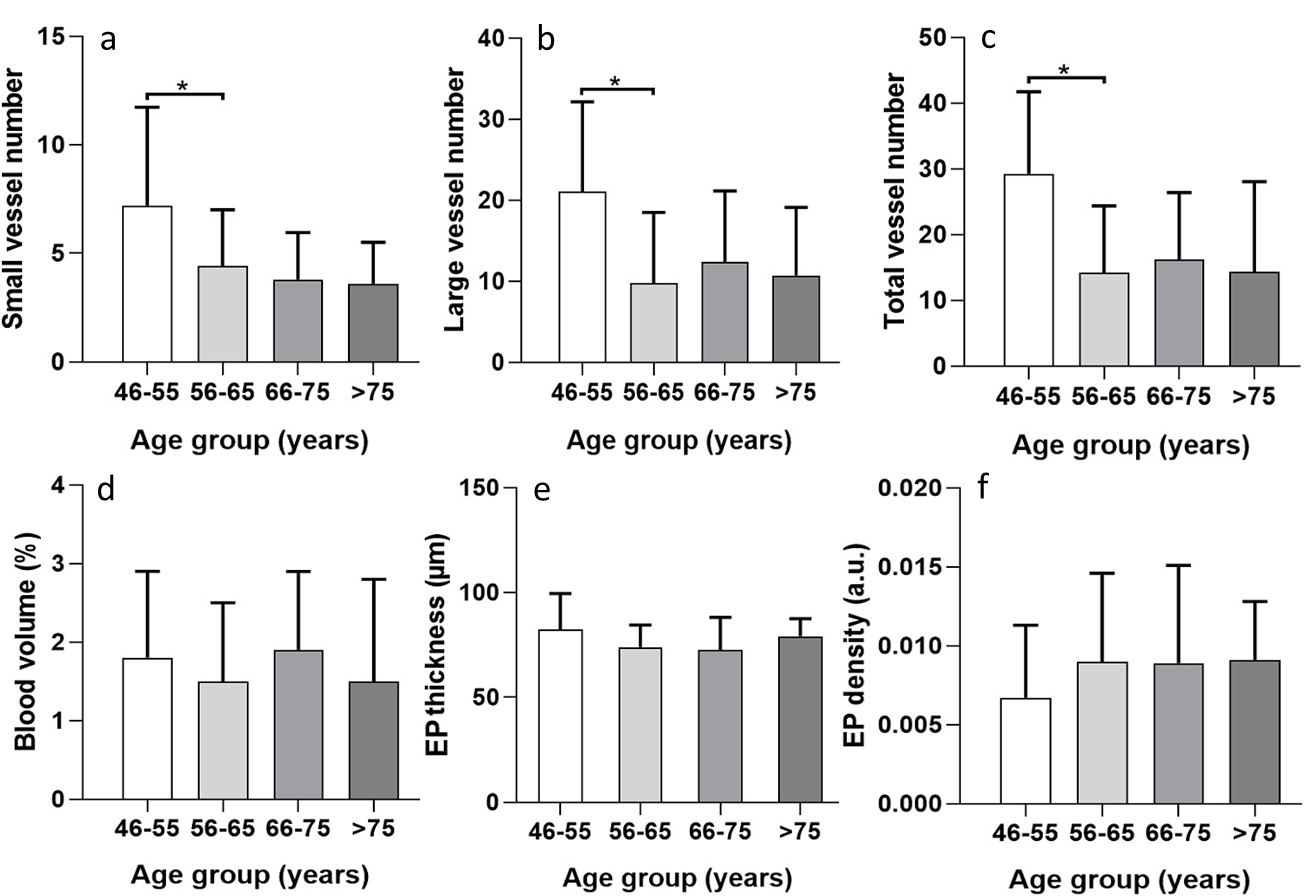


Figure S6. **Quantification of RSOM features in relation to patient ages**. 72 participants with diabetes from the first dataset were grouped based on their ages (years) as follows: 46-55 (n = 15), 56-65 (n = 18), 66-75 (n = 20), >75 (n = 10). Comparisons among age groups for six RSOM features were carried out: (a) Total number of small vessels (with diameter <= 40 µm) in DR layer; (b) Total number of large vessels (diameter > 40 µm) in DR layer; (c) Total number of vessels in DR layer; (d) Total blood volume in DR layer; (e) Thickness of EP layer; (f) Signal density of EP layer. * Represents P < 0.05. The numbers of small, large, and total vessels were significantly reduced for 45 to 55-year-old participants when compared to 56 to 65-year-old participants. The vessel number and blood volume decrease with increasing age, however, there is no significant change among the groups (56-65, 66-75 and >75). In addition, age does not obviously affect the Epidermal thickness and the Epidermal signal density. EP: Epidermis, DR: Dermis.


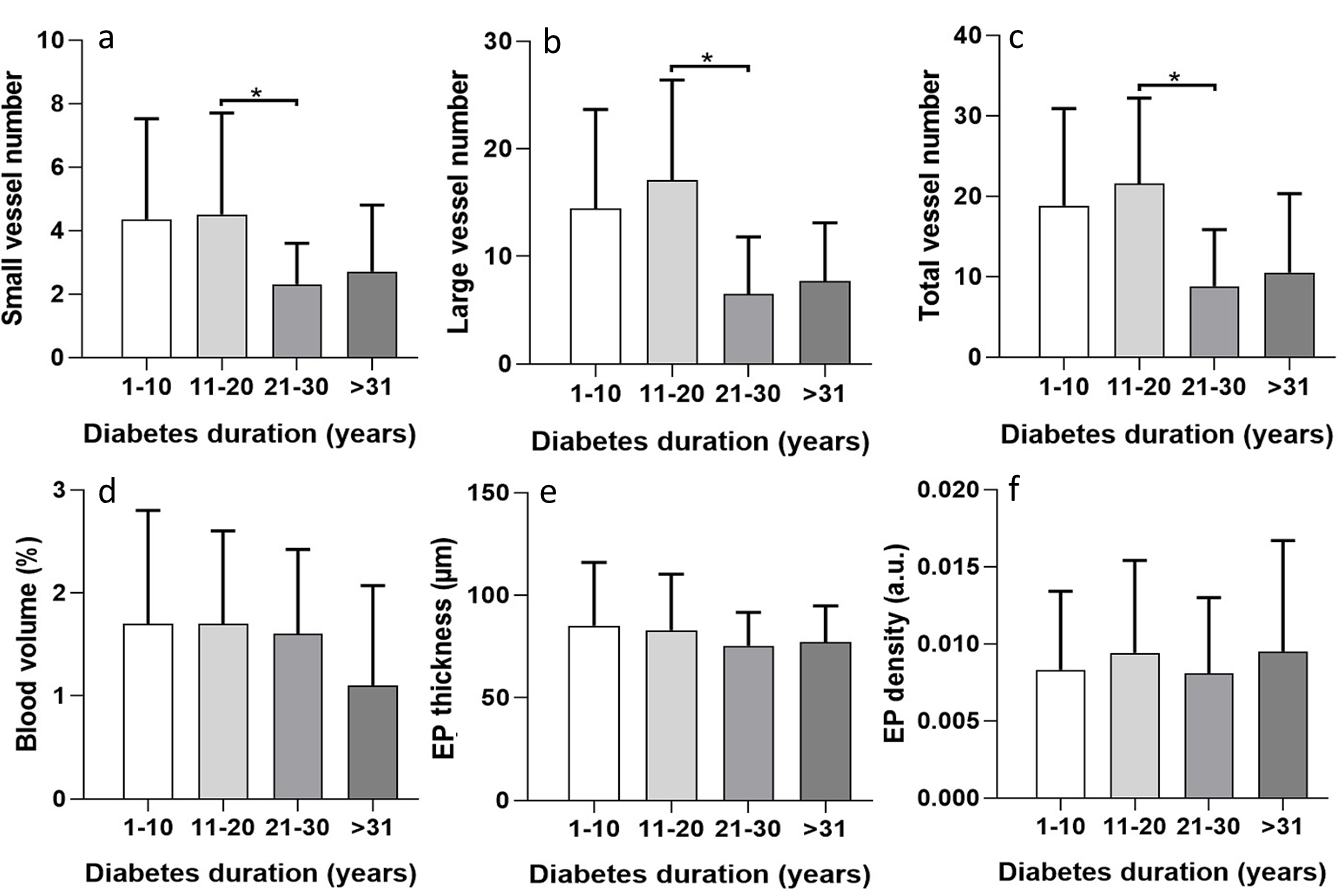


Figure S7. **Quantified RSOM features plotted against diabetes duration**. 72 participants with diabetes from the first dataset were grouped based on disease duration (years) as follows: 1-10 (n =25), 11-20 (n = 18), 21-30 (n = 10), >30 (n = 10). Comparisons amongst disease duration for six RSOM features were carried out: (a) Total number of small vessels (with diameter <= 40 µm) in DR layer; (b) Total number of large vessels (diameter > 40 µm) in DR layer; (c) Total number of vessels in DR layer; (d) Total blood volume in DR layer; (e) Thickness of EP layer; (f) Signal density of EP layer. * Represents P < 0.05. The numbers of small, large, and total vessels were significantly reduced in the group with 11 to 20 years of diabetes duration compared to the group with 21 to 30 years of diabetes duration. However, there were no significant changes when the other groups, i.e., 1-10 versus 11-20 or 21-30 versus >31 were compared. Mean blood volume reduced with the increment disease duration. The diabetes duration did not play a significant role in influencing the Epidermal thickness and the Epidermal signal density. EP: Epidermis, DR: Dermis.


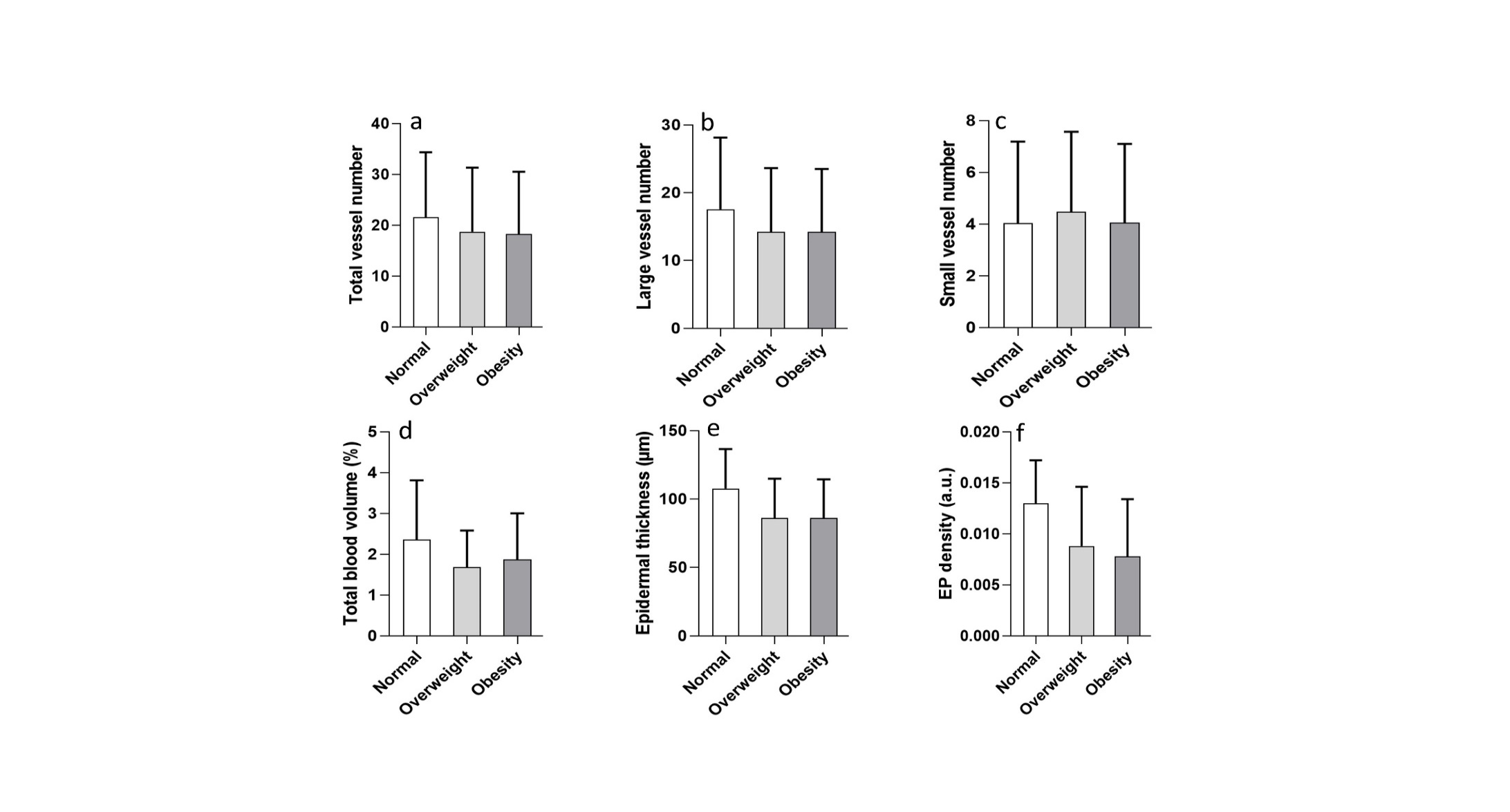


Figure S8. **Quantification of RSOM features in relation to body mass index (BMI).** 72 participants with diabetes from the first dataset were grouped based on BMI values as follows: Normal weight (18.5<BMI<25, n =18), Overweight (25<BMI<30, n = 25), Obesity (BMI>30, n = 20). Comparisons among the three BMI groups were done for six RSOM features: (a) Total number of small vessels (with diameter <= 40 µm) in DR layer; (b) Total number of large vessels (diameter > 40 µm) in DR layer; (c) Total number of vessels in DR layer; (d) Total blood volume in DR layer; (e) Thickness of EP layer; (f) Signal density of EP layer. * Represents P < 0.05. It can be observed that the mean numbers of total and large vessels were reduced between the group with normal weight and the overweight group. However, there is no significant difference. Mean blood volume, Epidermal thickness and Epidermal density were all decreased in the overweight and obesity groups compared to the normal weight patient group. However, the BMI values did not play a significant role in influencing RSOM biomarkers. EP: Epidermis, DR: Dermis.


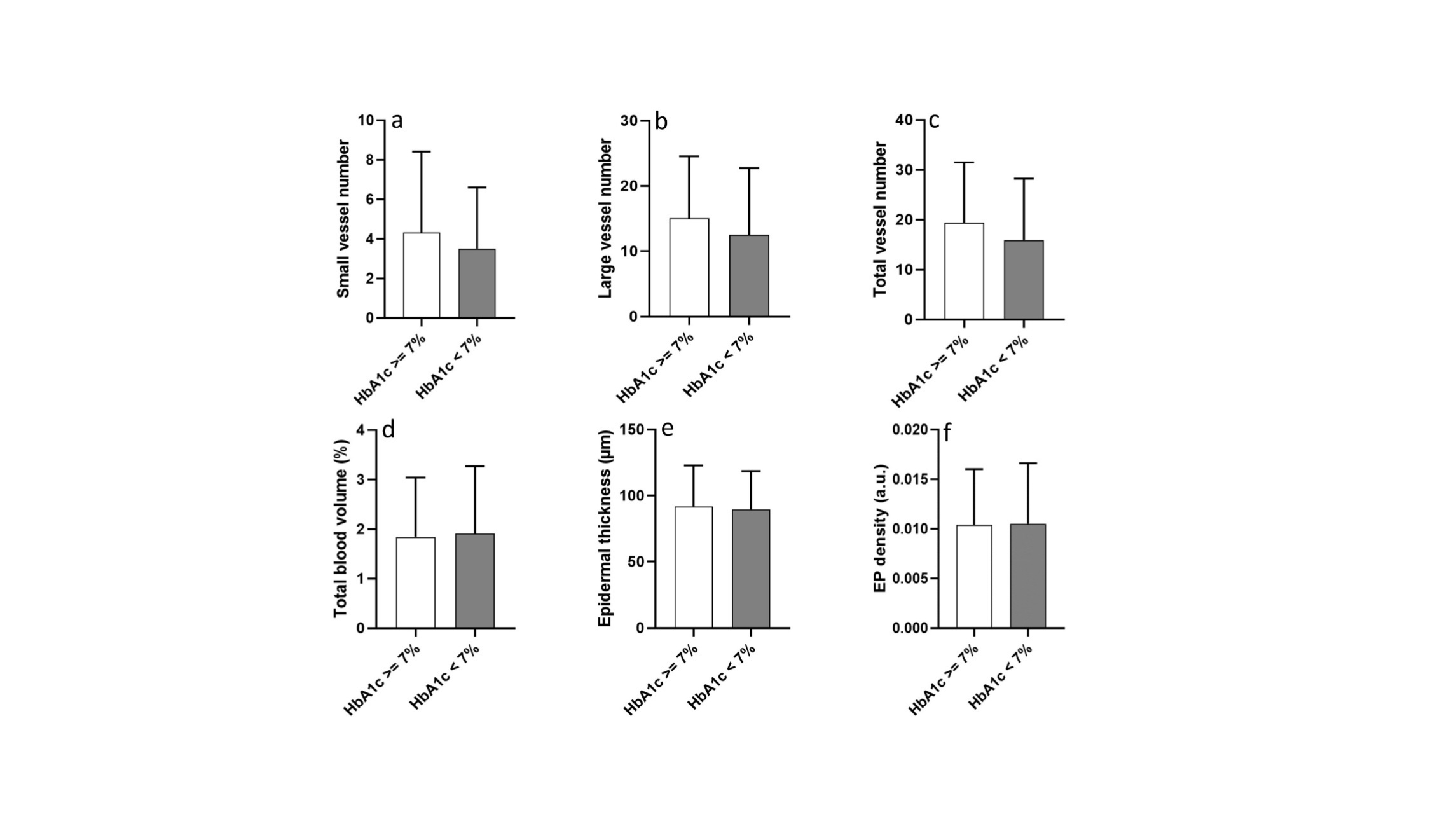


Figure S9. **Quantification of RSOM features associated with HbA1c values.** 72 participants with diabetes from the first dataset were grouped based on HbA1c values as follows: HbA1c >= 7% (n=34), HbA1c < 7% (n=38). Comparisons between the two HbA1c groups were carried out for six RSOM features: (a) Total number of small vessels (with diameter <= 40 µm) in DR layer; (b) Total number of large vessels (diameter > 40 µm) in DR layer; (c) Total vessel numbers in DR layer; (d) Total blood volume in DR layer; (e) Thickness of EP layer; (f) Signal density of EP layer. It can be noticed that the HbA1c values did not significantly affect the RSOM biomarkers examined. EP: Epidermis, DR: Dermis, HbA1c: Glycated hemoglobin.


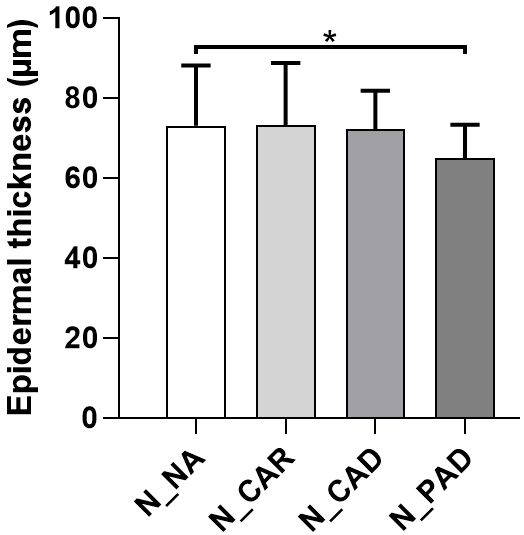


Figure S10. **Quantification of epidermal thickness in participants with diabetes with neuropathy and the presence of atherosclerosis**. Participants with diabetes were grouped as follows: N_NA (n=25), diabetic subjects with neuropathy and no atherosclerosis; N_CAR (n=7), diabetic subjects with neuropathy and carotid atherosclerotic disease; N_CAD (n=11), diabetic subjects with neuropathy and coronary artery disease; N_PAD (n=10), diabetic subjects with neuropathy and peripheral arterial disease; * Represents P < 0.05. The Epidermal thickness of the N_PAD group (64.89±8.43 µm) is significantly lower than in the group N_NA (73.02±15.08 µm).

**Classification between participants with diabetes and healthy volunteers based on RSOM biomarkers.** As demonstrated in Fig. 1 and 2, the RSOM biomarkers (Total blood volume and Small vessel number) were the two most indicative markers of diabetes (see Fig.1, 2), providing the starkest contrast between groups with or without diabetes. To investigate how RSOM biomarkers differentiate participants with diabetes from healthy volunteers, we trained a simple linear SVM (support vector machines) model to classify the participants with diabetes and the healthy volunteers. Since the RSOM data were collected from two hospitals and we simply separated them into the primary cohort (dataset 1 acquired from the first hospital: 72 participants with diabetes and 20 healthy volunteers) and the testing cohort (dataset 2 acquired from the second hospital: 23 participants with diabetes and 28 healthy volunteers). All patient information is listed in Table I and supplementary Table I. The total data (dataset 1 + dataset 2) based on the values of the two biomarkers were divided into four groups: non-diabetic volunteers from dataset 1 (NV-1, n=20), participants with diabetes from dataset 1 (DP-1, n=72), non-diabetic volunteers from dataset 2 (NV-2, n=28), participants with diabetes from dataset 2 (DP-2, n=23). As shown in Fig. S11a, the SVM classifier achieved 89.1% accuracy with 88.8% sensitivity and 90.0% specificity based on the biomarker of TBV (Total blood volume), while 90.2% accuracy with 93.1% sensitivity and 80.0% specificity was obtained using the biomarker of SVN (Small vessel number). Receiver operating characteristic (ROC) curves (Fig. S11b) were constructed based on the biomarkers of the TBV and SVN, revealing an area under the ROC curve (AUC) of 0.91 and 0.93 respectively. The combination of the two biomarkers resulted in a higher accuracy of 91.3% with 93.1% sensitivity and 85.0% specificity. Furthermore, we tested the classifier trained from the primary cohort on the testing cohort and achieved the prediction classification accuracy of 84.1% with 82.6% sensitivity and 82.1% specificity, which suggested that the SVM classifier determined by the two biomarkers performs well in classifying the participants of the testing cohort into the non-diabetic or the diabetic groups.


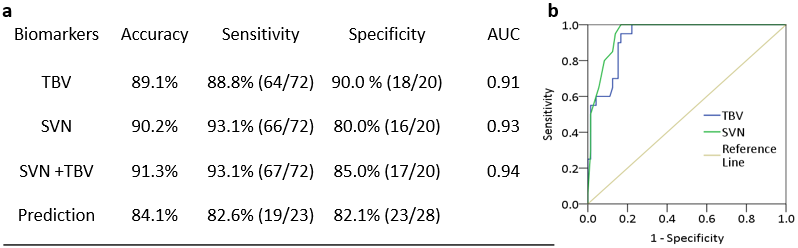


Figure S11. **Classification model to differentiate participants with diabetes and healthy volunteers**. The total data was grouped into a primary cohort (dataset 1, 72 participants with diabetes and 20 volunteers without diabetes) and a testing cohort (dataset 2, 23 participants with diabetes and 28 volunteers without diabetes). The total dataset was divided into four groups: non-diabetic volunteers from dataset 1 (NV-1, n=20), diabetic participants from dataset 1 (DP-1, n=72), non-diabetic volunteers from dataset 2 (NV-2, n=28), diabetic participants from dataset 2 (DP-2, n=23). The black line indicates the position of the linear SVM classifier based on the combination of the two biomarkers. a. Classification accuracy, sensitivity, specificity, and the AUC values computed by the linear SVM classifier on dataset 1. The trained SVM classifier was tested on dataset 2 with the prediction values. b. ROC plots in the differentiation of diabetes using TBV and SVN separately. TBV: Total blood volume, SVN: Small vessel number, ROC: Receiver operating characteristic, AUC: Area under the ROC curve.


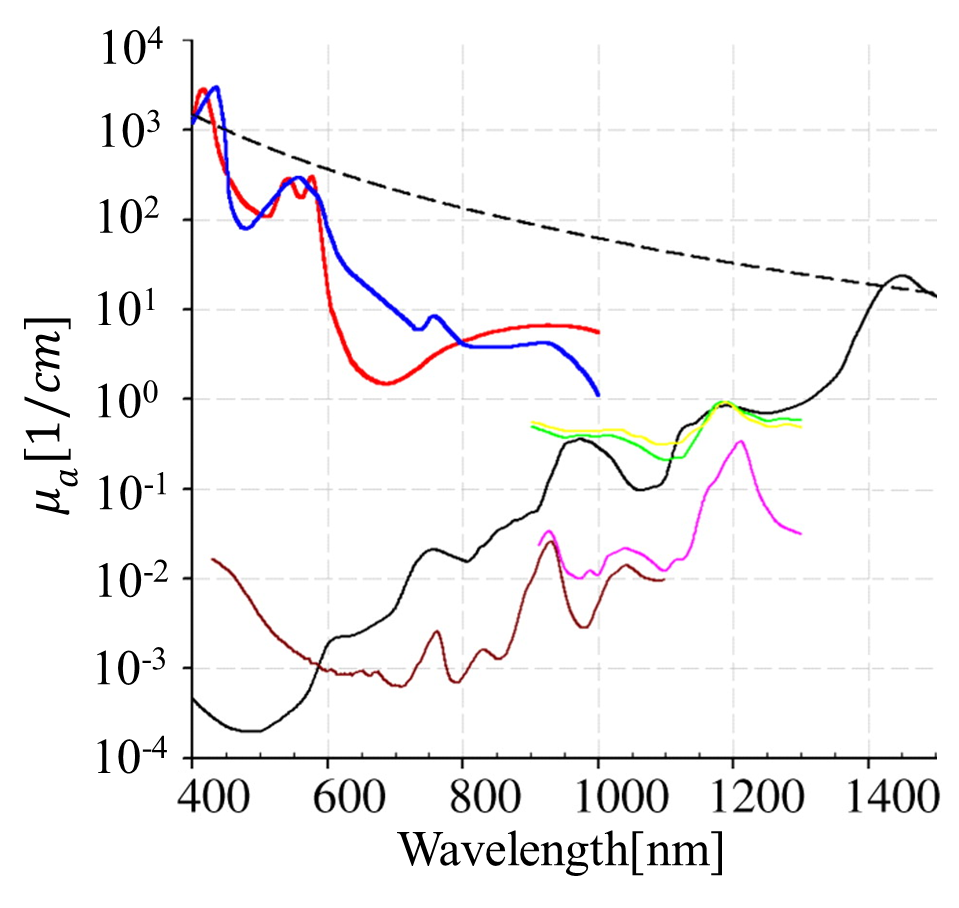


Figure S12. **Absorption coefficient spectra of several endogenous tissue chromophores.** Red line: oxyhemoglobin; blue line: deoxyhemoglobin; black line: water; brown line: lipid; black dashed line: melanin; green line: collagen: green line; and yellow line: elastin: yellow line. Data compiled by Scott Prahl, Oregon Medical Laser Center (http://www.omlc.ogi.edu/spectra).

Supplementary information accompanies the manuscript on the Light: Science & Applications website (<http://www.nature.com/lsa>)

**Bibliography**

1. Schwarz, M., Garzorz-Stark, N., Eyerich, K., Aguirre, J. & Ntziachristos, V. Motion correction in optoacoustic mesoscopy. *Sci Rep***7**, 10386 (2017).

2. Chiu, S.J.*, et al.* Automatic segmentation of seven retinal layers in SDOCT images congruent with expert manual segmentation. *Optics express***18**, 19413-19428 (2010).
